# Cytocompatible, Injectable, and Electroconductive Soft Adhesives with Hybrid Covalent/Noncovalent Dynamic Network

**DOI:** 10.1002/advs.201802077

**Published:** 2019-05-24

**Authors:** Yong Xu, Panagiotis A. Patsis, Sandra Hauser, Dagmar Voigt, Rebecca Rothe, Markus Günther, Meiying Cui, Xuegeng Yang, Robert Wieduwild, Kerstin Eckert, Christoph Neinhuis, Teuku Fawzul Akbar, Ivan R. Minev, Jens Pietzsch, Yixin Zhang

**Affiliations:** ^1^ B CUBE Center for Molecular Bioengineering Technische Universität Dresden Tatzberg 41 01307 Dresden Germany; ^2^ Helmholtz‐Zentrum Dresden‐Rossendorf Institute of Radiopharmaceutical Cancer Research Department of Radiopharmaceutical and Chemical Biology Bautzner Landstraße 400 01328 Dresden Germany; ^3^ Institute for Botany Faculty of Biology School of Science Technische Universität Dresden 01062 Dresden Germany; ^4^ Faculty of Chemistry and Food Chemistry School of Science Technische Universität Dresden Mommsenstraße 66 01062 Dresden Germany; ^5^ Helmholtz‐Zentrum Dresden‐Rossendorf (HZDR) Institute of Fluid Dynamics Bautzner Landstraße 400 01328 Dresden Germany; ^6^ Biotechnology Center Technische Universität Dresden Tatzberg 47/49 01307 Dresden Germany; ^7^ Leibniz Institute of Polymer Research Dresden (IPF) Max Bergmann Center of Biomaterials Dresden (MBC) Hohe Str. 6 01069 Dresden Germany

**Keywords:** 3D cell culture, adhesion, biocompatibility, PEDOT, small animal magnetic resonance imaging

## Abstract

Synthetic conductive biopolymers have gained increasing interest in tissue engineering, as they can provide a chemically defined electroconductive and biomimetic microenvironment for cells. In addition to low cytotoxicity and high biocompatibility, injectability and adhesiveness are important for many biomedical applications but have proven to be very challenging. Recent results show that fascinating material properties can be realized with a bioinspired hybrid network, especially through the synergy between irreversible covalent crosslinking and reversible noncovalent self‐assembly. Herein, a polysaccharide‐based conductive hydrogel crosslinked through noncovalent and reversible covalent reactions is reported. The hybrid material exhibits rheological properties associated with dynamic networks such as self‐healing and stress relaxation. Moreover, through fine‐tuning the network dynamics by varying covalent/noncovalent crosslinking content and incorporating electroconductive polymers, the resulting materials exhibit electroconductivity and reliable adhesive strength, at a similar range to that of clinically used fibrin glue. The conductive soft adhesives exhibit high cytocompatibility in 2D/3D cell cultures and can promote myogenic differentiation of myoblast cells. The heparin‐containing electroconductive adhesive shows high biocompatibility in immunocompetent mice, both for topical application and as injectable materials. The materials could have utilities in many biomedical applications, especially in the area of cardiovascular diseases and wound dressing.

## 1 Introduction

Hydrogels have drawn a lot of attention as promising extracellular matrix (ECM) mimicking biological materials. Various characteristics, such as swelling degree, water content, stiffness, porosity, viscoelastic properties, and biocompatibility can be tailored for different applications.^1^, ^2^, ^3^ The incorporation of electroconductive elements to hydrogels is an attractive design approach that combines the viscoelastic and mechanical properties of hydrogels with the conductivity of organic electronics.^4^, ^5^, ^6^, ^7^ Electroconductive hydrogels can be used in bioelectrical interfaces as bioresponsive electrodes, substrates that facilitate the electrical stimulation of cells or tissues (neuron or muscle), biosensors, implants, or for drug delivery under electrical stimulation.^8^, ^9^, ^10^ Conductive polymers, such as polypyrrole (PPy), polyaniline (PAni), and poly (3,4‐ethylenedioxythiophene) (PEDOT), have been used in the synthesis of electroconductive hydrogels due to their high conductivity and ease of processing.^11^, ^12^, ^13^, ^14^ However, the state‐of‐the‐art technology suffers from particular properties of conductive polymers, such as high hydrophobicity and insolubility, resulting in low adherence to wet substrates and poor penetration into living tissues.^5^, ^15^, ^16^ A highly hydrated electroconductive network, with biochemical and mechanophysical properties similar to the natural extracellular matrix, can provide a cell‐compatible scaffold for 3D cell culture.^17^ Moreover, a soft conductive adhesive, which can be utilized as injectable material, as well as bio‐ink for in situ bioprinting, will close the gap between electroconductive materials and biomaterials, lead to numerous medical and clinical applications.^18^, ^19^, ^20^

To incorporate new functions to a conductive polymer for biomedical utilities, there are several limits of “add‐on” approach because it does not alter the intrinsic properties of the electroconductive hydrogel network. For example, injectability, adhesion to biological tissues under wet conditions, and cell encapsulation are important for many biomedical applications but have proven to be very challenging. An attractive avenue is to develop self‐assembled physical electroconductive hydrogels.^13^ The noncovalent network can be disintegrated and re‐established, resulting in self‐healing and shear‐thinning properties important for developing injectable and printable materials.^21^, ^22^, ^23^ Moreover, noncovalently assembled electroconductive hydrogels are also particularly useful for cell encapsulation. Alternatively, recent work has shown that fascinating material properties can be realized with the bioinspired hybrid network, especially through the synergy between irreversible covalent crosslinking and reversible noncovalent interaction. For example, Guo and co‐workers designed a host–guest and covalent bonding‐based multifunctional conductive hydrogel with a desirable mechanical property and self‐healing ability and the resulting material can be used to build pressure‐dependent sensors.^12^ While the materials have shown high cytocompatibility for 2D cell culture, the covalent crosslinking reaction does not permit cell encapsulation in 3D hydrogel and completely impair the network dynamics mediated only by host–guest interaction.

Developing a cell‐compatible, injectable, and electroconductive soft adhesive is vital for the advancement of many biomedical emerging fields, such as artificial skins, adhesive wound‐healing patches, and injectable cardiac patches. However, a material with multiple functions cannot be easily realized with an add‐on approach and remains very challenging. In this work, we report a polysaccharide‐based electroconductive hydrogel system formed by a hybrid covalent/noncovalent dynamic network composed of aldehyde‐modified hyaluronic acid (HA‐ALD), glycol chitosan (GC), and PEDOT:Heparin (PEDOT:Hep) or PEDOT: poly(styrenesulfonate) (PEDOT:PSS). The dynamic network is mediated by noncovalent interactions (electrostatic, hydrogen bonding, π–π, and π–ion interactions), and dynamic covalent bonds (Schiff‐base formation). We aimed to develop a viscoelastic hydrogel that displays enhanced mechanical stability, as well as mechanical responsiveness, such as self‐healing, stress relaxation, shear‐thinning, and adhesion behavior. The biological functions of the electroconductive hydrogels were evaluated for their cytocompatibility for 2D/3D cell culture, effect on the induced myogenic differentiation of myoblast cells, and in vivo biocompatibility with immunocompetent mice both for topic application and as injectable materials.

## 2 Results and Discussion

**Figure 1:**
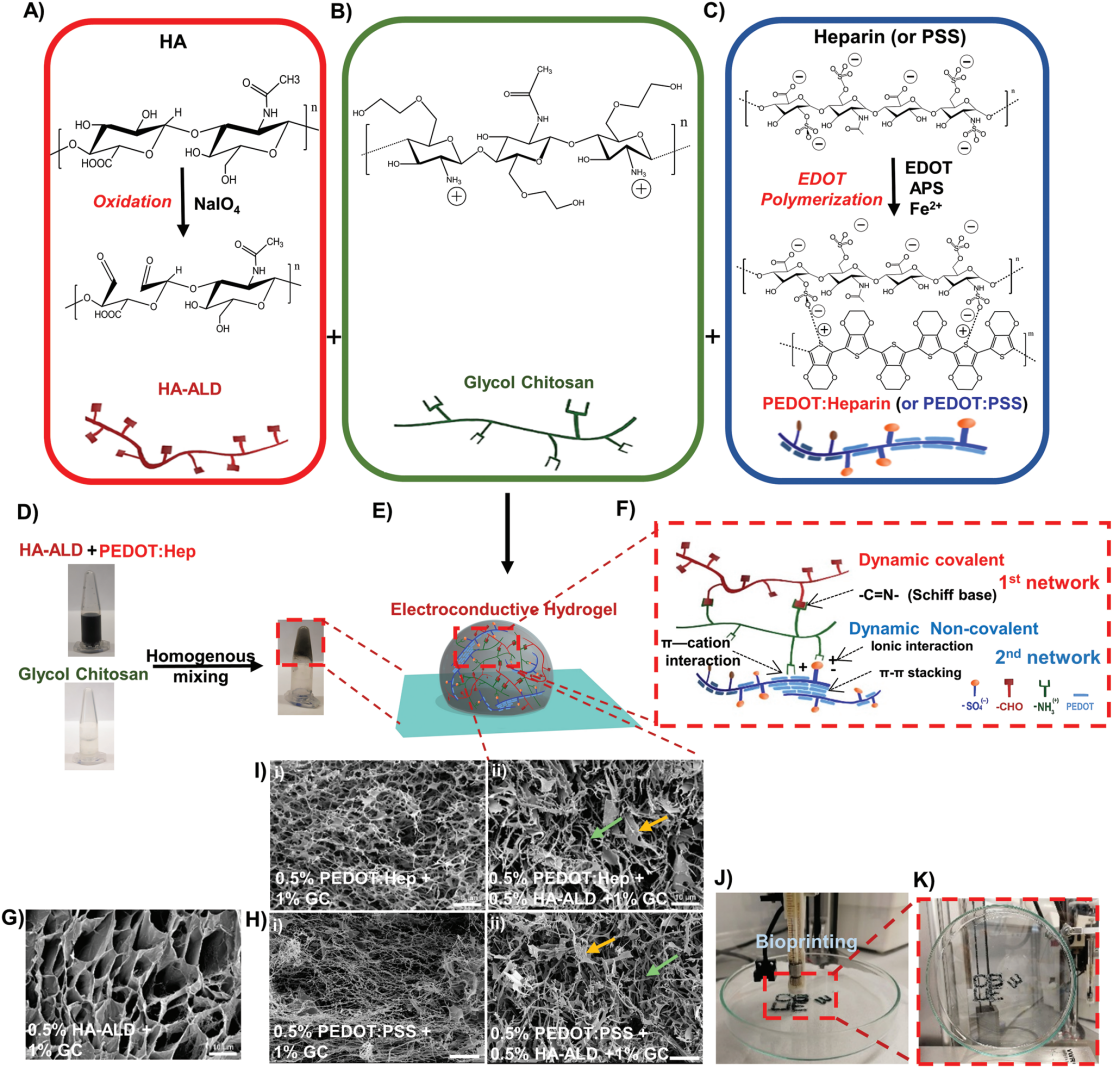
Characteristics of the dual cross‐linked electroconductive PEDOT:Heparin/HA‐ALD/GC hydrogels. A) Oxidation of HA by NaIO_4_, resulting in the formation of HA‐ALD with the characteristic presence of aldehyde groups. B) Structure of glycol chitosan. C) PEDOT:Hep and PEDOT:PSS synthesis by polymerizing EDOT to form PEDOT particles using heparin or PSS as dopant. The chemical structures of PSS and PEDOT:PSS are not illustrated. D) Representative hydrogel formation by homogeneous mixing of HA‐ALD + PEDOT:Hep with GC solutions in microtubes. E) Scheme of the 3D structure of the dual cross‐linked hydrogel network. F) Scheme of the interactions involved in the formation of the double network. G–I) Scanning electron microscopy (SEM) images of hydrogel networks. The arrows in the dual cross‐linked sample (I) indicate features, such as lamellar‐like structures (yellow arrow) or fibers (green arrow). Scale bar: 10 µm. J) Bioprinting of a PEDOT:Hep + HA‐ALD + GC hydrogel, which can adhere to the surface of a glass petri dish, and K) when holding the dish vertically.

We designed and synthesized polysaccharide‐based electroconductive hydrogels, which were formed by a hybrid network of aldehyde‐modified hyaluronic acid (HA‐ALD), glycol chitosan (GC), and PEDOT:Hep (or PEDOT:PSS) (**Figure** **1**A–C). Schiff base (imine) bond formation between HA‐ALD and GC resulted in reversible covalent crosslinking, while the noncovalent crosslinking was mainly mediated through the electrostatic interaction between negatively charged PEDOT:Hep or PEDOT:PSS and positively charged GC. Polysaccharides and PEDOT polymers were chosen not only because of their biocompatibility and electroconductivity, respectively, but also for introducing different types of noncovalent interaction to the dynamic matrix. The abundant hydroxyl groups of polysaccharides can provide a hydrophilic network as well as many hydrogen bond donors and acceptors. The PEDOT polymers were formed through π–π stacking between PEDOT nanoparticles and ionic interaction with the anionic polymer (Heparin or PSS). We speculated that the combination of covalent crosslinking and different noncovalent interactions can contribute to the adhesion to surfaces of various properties and cohesion within the network, which are both essential for an adhesive.

**Figure 2:**
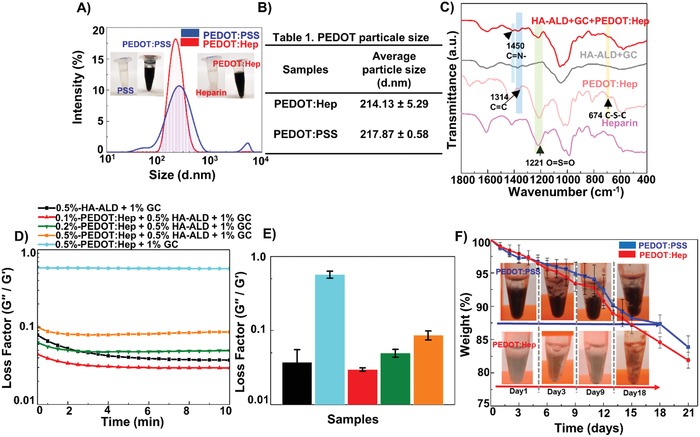
A) Particle size distribution of PEDOT:PSS and PEDOT:Hep solutions, determined by DLS. Inset: The solutions of PSS or heparin in MilliQ kept in microtubes and change from transparent to black after the polymerization of EDOT. B) The Z‐average particle size of PEDOT:Hep and PEDOT:PSS. Mean ± sd (*n* = 3). C) Fourier transform infrared (FT‐IR) spectra of Heparin, PEDOT:Heparin, HA‐ALD+GC and HA‐ALD+GC+PEDOT:Hep. D) PEDOT:Hep hydrogels (the loss factor plotted against the time). E) The loss factor at 10 min of gelation for hydrogels with PEDOT:Hep. The legend color is the same as Figure (D). F) Degradation assay of PEDOT:PSS or PEDOT:Heparin hydrogels. Inset: Images of PEDOT:PSS and PEDOT:Hep hydrogels during the degradation assay at 37 °C.

Aldehyde groups were introduced to HA through reaction with sodium periodate, as previously reported.^24^ Fourier transform infrared spectroscopy (FTIR) was used to confirm the reaction, with a newly formed peak at 1733 cm^−1^ corresponding to the stretching vibration of the C=O bond in HA‐ALD (Figure S3, Supporting Information). The polymerization of EDOT with Heparin or PSS as counterions was validated using ^1^H NMR and FTIR (**Figure** **2**C and Figure S1, Supporting Information). For example, for PEDOT:Hep the peaks at 1310 cm^−1^ and at 1520 cm^−1^ have emerged, corresponding to the C—C and C=C stretching vibrations of the quinoidal structure of the thiophene rings. The peaks at 1085 and 1144 cm^−1^ correspond to the C—O—C bond stretching in the ethylene dioxy ring. The peaks at 972, 830, and 681 cm^−1^ correspond to the C—S bond stretching vibrations in the thiophene ring.^25^ The particle sizes of conductive polymers can affect the rheological properties of self‐assembled hydrogels.^26^ We tuned the ratios between EDOT and sulfated polymer to control the particle sizes of different conductive polymers within a similar range. The particle sizes of PEDOT:Hep and PEDOT:PSS in water were characterized with dynamic light scattering (DLS), showing average sizes of 214.13 ± 5.29 and 217.87 ± 0.58 nm (mean ± sd, *n* = 3) respectively (Figure 2A,B). The UV–vis–NIR spectra of PEDOT:PSS and PEDOT:Hep exhibited high absorbance in the NIR a wavelength (700–1000 nm) typical for PEDOT materials (Figure S2, Supporting Information).^27^

The dynamic covalent hydrogel was formed by mixing HA‐ALD with GC, while dynamic noncovalent hydrogel was formed by mixing PEDOT:Hep or PEDOT:PSS with GC. Scanning electron microscope (SEM) image of the cross‐sections of the covalently cross‐linked hydrogel (HA‐ALD/GC) showed a porous network consisting of lamellar structures with large and smooth surfaces (Figure 1G). For the noncovalently cross‐linked hydrogel, SEM images displayed a dense network of fibers (Figure 1H). The hybrid dynamic electroconductive hydrogel was formed by adding GC to a premixed solution of PEDOT:Hep and HA‐ALD at room temperature (25 °C), resulting in dark‐blue hydrogels. The hybrid hydrogel with 0.5% PEDOT:Hep (Figure 1I), 0.5% HA‐ALD, and 1% GC showed a hybrid network possessing features from both the covalent and noncovalent hydrogels, with lamellar structures and fibers. This network is less dense and has a larger pore size than the noncovalent PEDOT:Hep/GC hydrogel (Figure 1G). Similarly, the hybrid hydrogel could also be formed using PEDOT:PSS (Figure 1H).

All three types of hydrogels were stable in aqueous solution, without detectable degradation over a time period of one month. Because of the biodegradability of HA (by hyaluronidase) and GC (by lysozyme), hydrogel degradation was tested by incubating the hybrid hydrogels in a mixture of both enzymes. As shown in Figure 2F, 17.5% and 18.5% of weight loss of the PEDOT:PSS/GC/HA‐ALD and PEDOT:Hep/GC/HA‐ALD hybrid hydrogels were measured after 21 d, respectively. The degradation was also visually apparent, as the intact hydrogels broke gradually into amorphous pieces. Interestingly, the PEDOT:Hep hybrid hydrogel became amorphous clusters, caused by the aggregation of degraded products.

**Figure 3:**
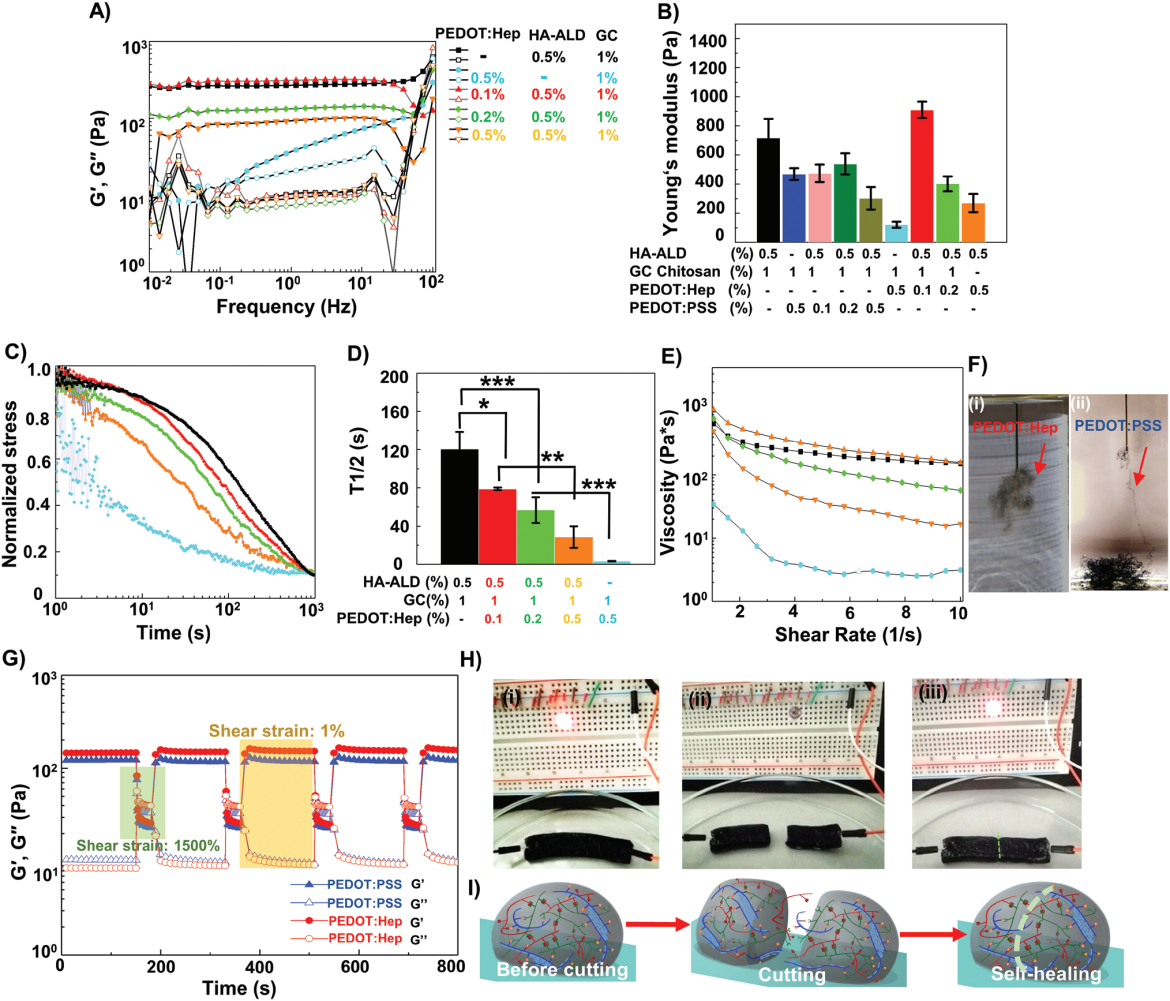
Rheological properties of the electroconductive hydrogels. A) Frequency sweep performed with a shear strain of 1% and the frequency increasing from 0.01 to 100 Hz. B) The Young's modulus of hydrogels at 10% shear strain (mean, error bar, *n* = 3). C) Stress relaxation curves of the hydrogels at a constant shear strain (1%). D) The stress relaxation half‐times (τ_1/2_) of the hydrogels (Statistically significant differences are shown with asterisks *p < 0.05, **p < 0.01, ***p < 0.001, mean, error bar, *n* = 3). E) Continuous flow experiments showing viscosity of the hydrogels plotted against the shear rate. F) The dual cross‐linked hydrogels were syringe‐injectable and thin filaments (indicated by the arrows) were be extruded into PBS solution. G) Self‐healing property of the hydrogels when the alternate step strain was switched from 1% to 1500%, indicated by the recovery of the elastic modulus. H) The self‐healing of the hydrogel highlighted by an electric circuit with an LED. (i) The electroconductive hydrogel closed the circuit and the LED lit up. (ii) The hydrogel was cut into two pieces and the circuit opened. (iii) The hydrogel pieces were placed together and left to self‐heal into one block (after 5 min) and the circuit was closed, as indicated by the LED. I) Scheme of the self‐healing process of the electroconductive hydrogel.

The gelation of hydrogels was evaluated by measuring the storage (*G*′) and loss (*G*′′) moduli over time. The covalent, noncovalent, and hybrid hydrogels exhibited fast gelation (Figure 2D). As compared to the covalent hydrogel, both noncovalently cross‐linked hydrogels (PEDOT:PSS/GC and PEDOT:Hep/GC) showed higher loss factors (δ = *G*′′/*G*′), indicating that the hydrogels were more viscous (Figure 2E). Incorporating dynamic covalent crosslinking (through adding HA‐ALD) into the network led to lower δ value. As the amino group of GC can either form a neutral imine bond with HA‐ALD or form electrostatic interaction with heparin or PSS, reducing the PEDOT:PSS or PEDOT:Hep content further increased the elastic portion (**Figure** **3**A) and Young's modulus (Figure 3B), as the dynamic covalent crosslinking became more dominant in the network.

The reversible imine bond led to a dynamic network and the HA‐ALD/GC hydrogel showed stress relaxation property with a *T*_1/2_ of 120 s (Figure 3C,D), while the irreversibly crosslinked hydrogels normally exhibited *T*_1/2_ > 2000 s.^28^, ^29^, ^30^ As expected, incorporation of noncovalent crosslinking accelerated the network dynamics and stress relaxation, as shown by the gradual reduction of *T*_1/2_ upon increasing the PEDOT:Hep concentration. The full noncovalent PEDOT:Hep/GC hydrogel possessed the lowest *T*_1/2_ of 2 s. The hydrogels also showed the non‐Newtonian behavior of shear thinning. As expected, with the increase of PEDOT:Hep concentration in the hybrid network, the shear‐thinning behavior became more remarkable (Figure 3E). The hybrid materials exhibited the rheological properties associated with the dynamic network, while the loss factor (*G*´´/*G*´), stress relaxation, and shear‐thinning could be tuned by varying the ratio between the two types of crosslinking, as the covalent and noncovalent bonds possessed different bond energy.

Self‐healing property of hybrid hydrogels was tested by performing step‐strain measurements (Figure 3G). At high strain of 1500% both hybrid hydrogels yield, with *G*' and *G*” showing rapid decrease and at low strain of 1%, the hydrogels rapidly recovered to their original state. This behavior was stable over many cycles, demonstrating the stable self‐healing ability of the hybrid network. Interestingly, under 1500% shear strain, the loss factors were increasing over the cycles (Figure S20, Supporting Information), reflecting enhanced shear‐thinning property. We speculate that because the dynamic network is reformed in each self‐healing cycle, the viscosity of the initial pre‐gel solution can be affected by the network rearrangement under very high shear stress treatment. The self‐healing property can also be highlighted by an electric circuit experiment containing the hybrid PEDOT:Hep hydrogel (Figure 3H). The hydrogel was cut into two pieces. The LED light could fully recover after putting the two pieces in proximity without applying extra force. The shear‐thinning and self‐healing hybrid conductive hydrogels could be easily extruded through a microsyringe needle with an inner diameter of 200 µm (Figure 3F, Movies S1–S3, Supporting Information). Moreover, the materials could also be printed on glass and tissue using a 3D microextrusion printer (custom‐made) (Figure 1J,K, Movies S4 and S10, Supporting Information). Injection under biological wet conditions is extremely attractive for many biomedical utilities. Therefore, we tested the injection of the hybrid hydrogels into aqueous solution (Movies S1 S2, Supporting Information). The extruded filaments remained intact. Strikingly, the filaments of hybrid PEDOT:Hep hydrogel stuck to each other and formed an entangled cluster, while the PEDOT:PSS filaments remained separately and sunk to the bottom of the test vessel filled with water. This surprising observation suggested that the hybrid PEDOT:Hep hydrogel could fulfill the requirements not only for underwater injection but also for biological tissue‐relevant wet surfaces adhesion.

**Figure 4:**
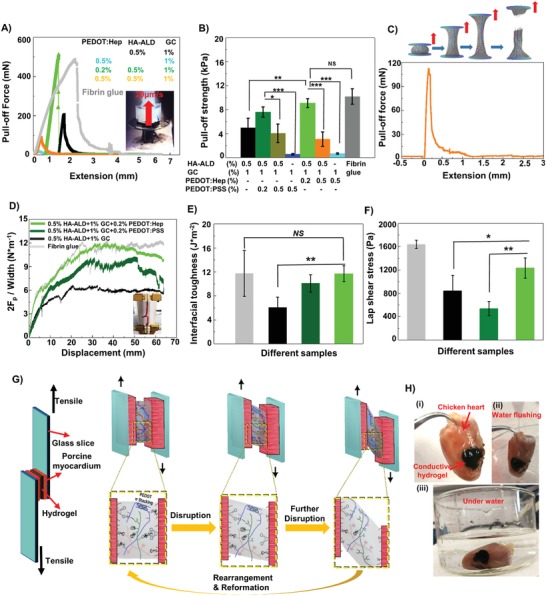
The adhesive property of electroconductive hydrogel. A) Adhesion pull‐off testing of PEDOT:Hep hydrogels, where the adhesive strength is plotted against the extension. Inset: Detail of the experimental setup where the hydrogel adhered between two glasses that were pulled apart at a constant speed. B) The adhesive strength of the hydrogels. (Statistically significant differences are shown with asterisks *p < 0.05, **p < 0.01, ***p < 0.001, mean, error bar, n = 3). C) Scheme of the adhesion measurement, which shows the behavior of the hydrogel as the extension increases in correspondence with the adhesion force curve plotted against the extension (0.5% PEDOT:Hep 0.5% HA‐ALD 1% GC). D) Double peeling force per band‐shaped adhesive width obtained while peeling on porcine myocardium tissue band from the other glued by the different conductive hydrogels. (Inset: Photo of the double peeling test setup). E) The calculated interfacial toughness corresponding to (D) (n = 4). Statistically significant differences are shown with asterisks *p < 0.05, **p < 0.01, ***p < 0.001, mean, error bars, n = 4. F) lap‐shear strength of hydrogels and commercial fibrin glue sheared on flat porcine myocardium tissue (n = 4). G) Image of the lab‐shear experimental setup and adhesion mechanism of hybrid covalent/non‐covalent dynamic network in conductive hydrogels. H) (i) Adhesion of the electroconductive hydrogel (0.5% PEDOT:Hep 0.5% HA‐ALD 1% GC) on muscle tissue (chicken heart), (ii) the hydrogel remains adhered to the muscle tissue under flushing with water and (iii) under water.

The adhesive properties of the hydrogels were investigated by the use of pull‐off tests, measuring the maximum force at the peak of the force–distance curve (Movies S5 and S6, Supporting Information). The noncovalent hydrogels showed weak adhesive strength <1 kPa, while the dynamic covalent hydrogel GC/HA‐ADL exhibited remarkably enhanced adhesiveness of 4.9 kPa (**Figure** **4**A,B). Interestingly, by adding PEDOT:PSS or PEDOT:Hep to the GC/HA‐ADL hydrogel, the resulting hybrid hydrogels exhibited the highest adhesive strength. Further increasing the PEDOT:PSS or PEDOT:Hep concentration caused reduced adhesiveness. The pull‐off strength measurements are in good agreement with the elastic moduli obtained from tensile tests (Figure S16, Supporting Information), which measure the cohesion strength of materials. Similarly, increasing the noncovalent proportion of the network by increasing PEDOT:PSS or PEDOT:Hep concentration also reduces the cohesion strength. Shifting the hybrid network into the direction of noncovalent crosslinking caused weakened cohesion (Figure 4B,C), as these hydrogels have also shown reduced storage modulus. The gelation process involves the formation of the double network including dynamic covalent and dynamic noncovalent networks. As the amino group of GC can either form a neutral imine bond with HA‐ALD or form electrostatic interaction with heparin or PSS, increasing the PEDOT:PSS or PEDOT:Hep content reduces the covalent portion of the network. Thus, these two dynamic interactions will affect both the gel–substrate surface interaction (surface adhesion) and the mechanical strength of gels (cohesion), while neither covalent hydrogel nor noncovalent hydrogel shows high adhesiveness. In comparison to adhesion under dry conditions, a fully hydrated substrate prevents the contact (adhesion) of hydrogel associated with the noncovalent interaction. Therefore, it is notoriously difficult to develop underwater adhesives based on hydrogels. In addition to the observation from the underwater injection and the pull‐off measurements, manual handling of the materials also suggested that the hybrid PEDOT:Hep hydrogel was the most adhesive material of all our fabricated and tested hydrogels. Polysaccharides have been found in many adhesive materials, also including natural ones,^31^, ^32^, ^33^ as they present a rich source of hydrogen bond donors and acceptors. By replacing the hydrophobic polystyrene backbone with polysaccharide chain, the hybrid PEDOT:Hep hydrogel allowed for more contact adhesion to highly hydrated surfaces.

The adhesive properties were additionally characterized using peeling tests and lap‐shear (Figure 4D–F) with hydrogels on porcine myocardium tissues. In both tests, clinically used fibrin glue and hybrid PEDOT:Hep/GC/HA‐ALD hydrogel showed similar adhesive performance, which is remarkably higher than that of hybrid PEDOT:PSS/GC/HA‐ALD hydrogel or GC/HA‐ALD hydrogel. We have also performed repeated adhesion tests with lap‐shearing of the same sample (Figure S15, Supporting Information). After three cycles, a moderate loss of adhesion strength was observed for hybrid PEDOT:Hep/GC/HA‐ALD hydrogel. As shown in Figure 4G, electrostatic interactions, hydrogen bonds, hydrophobic interactions, and Schiff‐base formation occur in the hydrogel network, as well as at the interface between the substrate (e.g., mammal skin tissue) and hydrogel. The successive disruption, rearrangement, and reformation of the network when pulling a viscoelastic bulk material result in the adhesive property, as all the reactions are dynamic and reversible during several repeated cycles of attachment and detachment. Because of the difference in bond energy, to break the dynamic imine covalent bond is more difficult than disrupting the noncovalent interactions. Because the stability of an adhesive in aqueous environment is very important for its clinical suitability, we also measured hydrogel shear stress of hydrated samples. As shown in Figure S17 (Supporting Information), both fibrin glue and hybrid PEDOT:Hep/GC/HA‐ALD hydrogel show a moderate decrease of adhesive strength after immersing the materials in water for 6, 12, and 24 h. The reduction in shear stress after 6 h was mainly caused by the fact that the tissues swelled and became fragile after the long treatments in aqueous solution. After either the reversible test or underwater treatment, the hybrid PEDOT:Hep/GC/HA‐ALD hydrogel still possesses enough adhesive strength (>800 Pa in the lap shear test on porcine myocardium) for biomedical applications.

The underwater adhesion of the hybrid PEDOT:Hep hydrogel to tissues was further evaluated after gelling in situ on the surface of the chicken myocardium (Figure 4H‐i–iii). After rinsing with water and floating in a water bath with strong stirring (240 rpm min^−1^), the hydrogel remained intact and adhered tightly to the chicken heart tissue without detachment, suggesting a considerable potential for applications in vivo. Remarkably, the excellent binding strength of the material was able to withstand heavy water flushing of hydrogel on the tissue (Figure 4H; Movies S7 and S8, Supporting Information), suggesting that the hydrogel as a potent adhesive for a wide range of applications.

**Figure 5:**
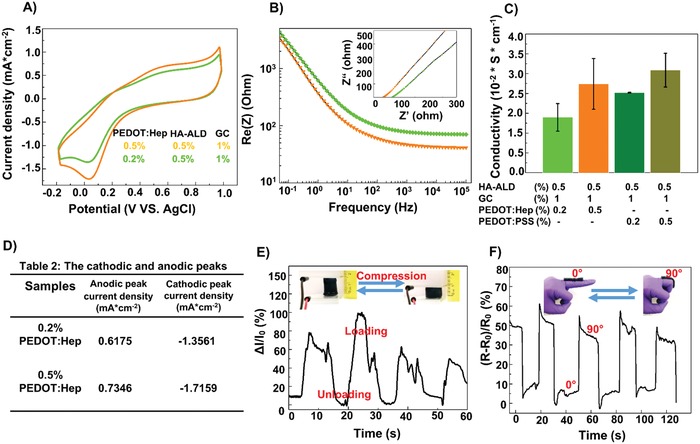
Electrochemical characteristics of the dual cross‐linked electroconductive hydrogels. A) Cyclic voltammograms (current density vs the potential) of hydrogels with different concentrations of PEDOT:Hep, in PBS at a scan rate of 50 mV s^−1^. B) Electrochemical impedance spectroscopy (*Z* vs frequency) of hydrogels with different concentrations of PEDOT:Hep (inset: Nyquist plot of the hydrogels). C) The conductivity of hydrogels (means, error bars, *n* = 3). D) The current density at the cathodic and anodic peak of the voltammograms of hydrogels with different concentrations of PEDOT:Hep. E) The relative current variation as function of time under a continuous cyclic tensile loading−unloading. Inset: Images of the compression and relaxation processes of the hydrogel, which undergoes a rapid recovery of its original shape to its a compressive deformation. F) Changes in the relative resistance during the bending of a finger at of angles at 0° and 90°.

The hydrogels were electrochemically studied in a three‐electrode system. The hydrogels showed a stable electrochemical behavior after many cycles (>10) of voltage ramping. It is notable that the cyclic voltammetry (CV) curves of the hybrid PEDOT:Hep or PEDOT:PSS hydrogels were typical of PEDOT containing polymers (**Figure** **5**A), showing the characteristic oxidation and reduction peaks. The hybrid PEDOT:Hep hydrogels showed an anodic peak (Epa) at ≈0.63 V and a cathodic peak (Epc) at 0.03 V. Increasing the concentration of PEDOT:Hep from 0.2% to 0.5% resulted in the increase of the current at both peaks. In the case of PEDOT:PSS hydrogels, the Epa was observed at 0.65 V and the Epc at 0.02 V. Increasing the PEDOT:PSS concentration from 0.2% to 0.5% also caused enhanced current at both peaks (Figure 5D). The electrochemical impedance spectroscopy (EIS) was used to confirm the electrical performance of the materials. The EIS is presented as Nyquist plots (Figure 5B). Upon increasing the concentration of conductive polymer (either PEDOT:Hep or PEDOT:PSS), reduced impedance for all the frequencies was measured. The conductivity of hybrid PEDOT:PSS hydrogel was slightly higher than the hybrid PEDOT:Hep hydrogel when same concentrations of PEDOT polymer were used (Figure 5C).

Owing to the hydrogels' adhesiveness, electrical conductivity, and self‐healing capability, we investigated their response to deformation, for the potential applications as sensors in wearable devices and implants. As illustrated in Figure 5E, we measured the response of conductivity to loading/unloading cycles. Compressing the hybrid PEDOT:Hep hydrogel resulted in an instant increase of measured current, while the change was reversible immediately after removing the stress. In another experimental setup (Figure 5F), hybrid PEDOT:Hep hydrogel was attached to a trigger finger covered by gloves, in order to monitor finger movements electronically. Bending the finger at angles from 0° to 90° caused stretching of the hydrogel, which led to instant increase in resistance, while the change could be reversed immediately after moving back to the original position.

The hybrid PEDOT:Hep hydrogel and PEDOT:PSS hydrogel exhibited various features associated with the dynamic network, to be printable and injectable, possessing stress relaxation and self‐healing properties, while PEDOT and polysaccharides led to electroconductivity and degradability, respectively. The hybrid network also generated new material properties. The hydrogels were mechanically stable, remained intact over 10 cycles of compression or bending, as well as water flushing. In contrast, both the noncovalent PEDOT:polymer/GC hydrogel and the covalent GC/HA‐ALD hydrogels were more fragile and broke easily into small pieces. Moreover, the hybrid materials have shown strong adhesion to various surfaces, including animal tissues. As their stiffness could match that of soft tissues, we investigated their cytocompatibility for their potential clinical applications, such as adhesives or implants. Strong adhesion requires not only noncovalent and covalent bonding but also physical interpenetration, thus involving interfaces with cells in both 2D and 3D. Therefore, we investigated the culture of myoblast cells either encapsulated in the soft conductive adhesives or seeded on the surfaces.

**Figure 6:**
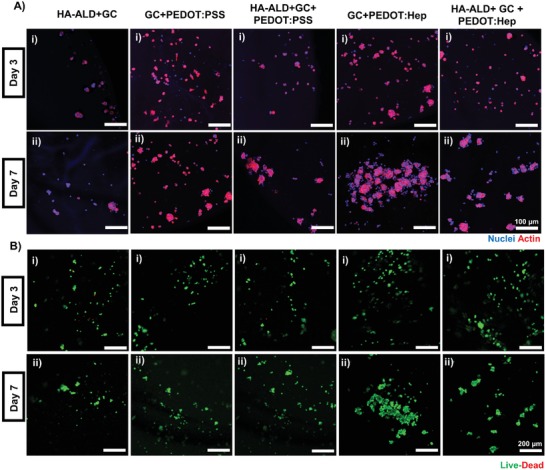
LSM images of electroconductive hydrogel induced myogenesis of C2C12 myoblast cells. A) Immunofluorescence staining of the cells at day 3 and day 7 of the nuclei (DAPI) and actin (phalloidin). B) Live–dead staining of mouse myoblasts cultured in hydrogels at day 3 and day 7, with Calcein‐AM (viable) and propidium iodide (dead).

Mouse skeletal myoblast line C2C12 cells were embedded in dynamic covalent hydrogel GC/HA‐ALD, noncovalent hydrogels PEDOT:PSS/GC and PEDOT:Hep/GC, and hybrid PEDOT:PSS and PEDOT:Hep hydrogels. The covalent hydrogel led to the lowest cell growth, while the cells embedded in noncovalent PEDOT:Hep/GC hydrogel exhibited the highest cell density after culturing for 7 d (**Figure** **6**A). Interestingly, the myoblasts formed large clusters in the heparin‐containing hydrogels, especially in the noncovalent PEDOT:Hep/GC hydrogel. Cell viability was assessed by live/dead cell staining 3 and 7 d after the set up (Figure 6B). All hydrogels exhibit high cytocompatibility, while a few cell deaths could be observed in the covalent GC/HA‐ALD hydrogel.

**Figure 7:**
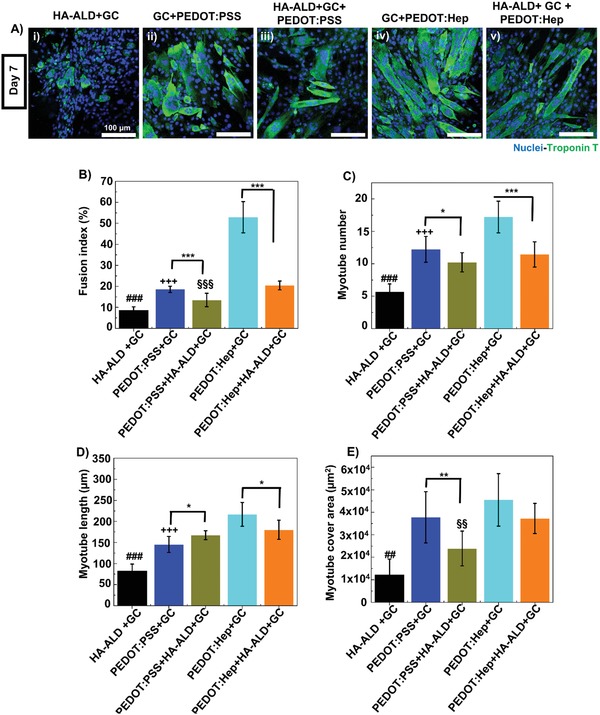
Electroconductive hydrogel induced myogenesis of C2C12 myoblast cells. A) Troponin T (TnT) and nuclei immunofluorescence staining of mouse skeletal myoblasts at day 7 of differentiation. Scale bar: 100 µm. B–E) Morphometric parameters of C2C12 cells at day 7 of differentiation on the hydrogels and quantified with ImageJ software. B) Fusion index. C) Myotude number. D) Myotube length. E) Myotube cover area. Statistically significant differences found for HA‐ALD + GC hydrogel with other electroconductive hydrogels (###) *p* < 0.001. Statistically significant differences were also found between PEDOT:PSS + HA‐ALD + GC and PEDOT:Hep + HA‐ALD + GC for (§§§) *P* < 0.001, (§§) *P* < 0.01. Columns represent means and error bar standard deviation (*n* = 9).

C2C12 cells could also adhere and proliferate on all types of hydrogels. Upon subjected to differentiation condition, myogenesis into mature and multinucleated myotubes was evaluated by immunofluorescence staining for the myogenic differentiation marker troponin T. Remarkable differences in terms of myogenic differentiation were observed among the myoblasts cultured on different substrates. The C2C12 myoblast cells seeded on noncovalent hydrogel films differentiated into multinucleated myotubes, revealing tube‐like morphology.^34^ On covalent nonconductive GC/HA‐ALD hydrogel, differentiated cells did not show myotube‐like morphology (**Figure** **7**A). Interestingly, cells differentiated into mature myotubes with a long myotube‐like morphology on conductive hydrogels. Fusion index, troponin T‐positive area, and myotube length were determined with ImageJ software (Figure 7B–E). The fusion index was significantly higher when the C2C12 myoblasts were seeded on the noncovalent PEDOT:Hep/GC hydrogel, as compared with other materials. The fusion index agreed quite well with the calculated myotube number and length as well as with the troponin T‐positive area. For both noncovalent and hybrid hydrogels, heparin‐containing materials led to a higher fusion index as compared to the PSS‐containing materials. While conductive polymer, such as PEDOT, cannot be avoided to generate electroconductive matrix, by replacing PSS with heparin as counterion in the PEDOT synthesis, the PEDOT:Hep material could probably provide a more ECM‐like environment for myogenesis by maximizing the polysaccharide content.

To investigate the biocompatibility of the electroconductive soft adhesives, we studied the hydrogels either as injectable materials or for topical application with immunocompetent mice. The hybrid PEDOT:Hep hydrogel extruded from a syringe adhered very well on mouse skin (Figure S11A, Supporting Information). When the mouse was running in the cage, the hydrogel touched and stuck to the wall, and was then torn apart by the mouse, as the electroconductive hydrogel also has very good adhesive property to plastic surface (Figure S11B and Movie S9, Supporting Information). This was observed several times in a time period of 30 min. After that, the material was removed by a tweezer, no adverse skin reaction occurred in the following 2 weeks.

**Figure 8:**
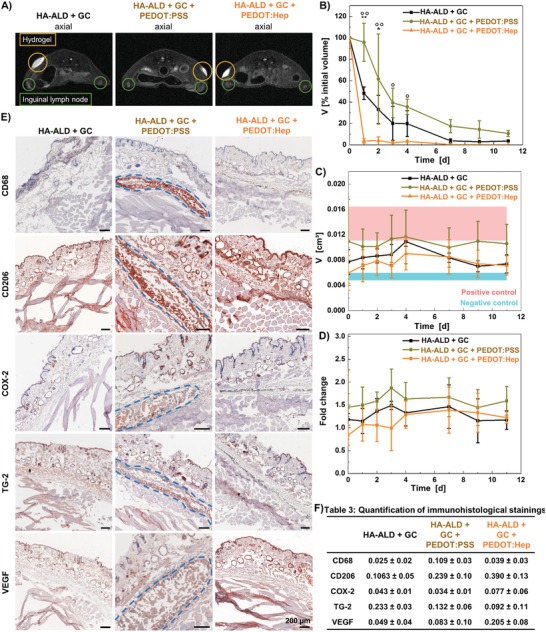
In vivo evaluation of hydrogel degradation and biocompatibility. A) Localization of hydrogels and inguinal lymph nodes by MRI. B) Volume determination of hydrogels by MRI, */° *p* < 0.05; **/°° *p* < 0.001; *n* = 3; Mean ± SD; one‐way ANOVA, Bonferroni post‐hoc test, * HA‐ALD + GC versus HA‐ALD + GC + PEDOT:PSS, HA‐ALD + GC + PEDOT:Hep; ° HA‐ALD + GC + PEDOT:PSS versus HA‐ALD + GC + PEDOT:Hep. C) Volume determination of inguinal lymph nodes at hydrogel injection site by MRI, compared to negative (untreated) and positive (TPA injection) control, *n* = 3; Mean ± SD; one‐way ANOVA, Bonferroni post‐hoc test. D) Comparison of volumes of lymph nodes at the injection site compared to site without hydrogel injection. E) Representative immunohistological images of markers for pan‐macrophages (CD68), M2 macrophages (CD206), inflammation (COX‐2), matrix remodeling (TG‐2), and angiogenesis (VEGF), cell nuclei in blue and positive immunohistological staining in red, blue line indicates hydrogel–tissue interface. F) Quantification of immunohistological stainings (positively stained area related to cell nuclei area).

GC/HA‐ALD hydrogels, hybrid PEDOT:PSS hydrogels and hybrid PEDOT:Hep hydrogels were injected subcutaneously in immunocompetent nude mice, in groups of three mice each (Figure S18, Supporting Information). Degradation in vivo represents an important parameter to evaluate the bioadhesives. While a quickly degradable material can be used as temporary adhesive, slow degradation rate would suggest its application for long‐term uses. We applied magnetic resonance imaging (MRI) to follow the degradation (**Figure** **8**A,B). Interestingly, the degradations of hydrogels in vivo were remarkably faster than those observed in the in vitro experiments (Figure 2F). Hybrid PEDOT:Hep hydrogel degraded quickly, with only 4% of the initial volume left 1 d after the injection and full degradation after 7 d. Hybrid PEDOT:PSS hydrogel degraded remarkably slower, while 10% of the initial volume remained in the tissue after 11 d. Degradation of GC/HA‐ALD hydrogel was slower than that of hybrid PEDOT:Hep hydrogel and faster than that of hybrid PEDOT:PSS hydrogel. For all hydrogels, the degradations are faster than the observed degradation by lysozyme and hyaluronidase in vitro. This could be explained by the presence of heparinase in the skin,^35^, ^36^ which is able to degrade the heparin component in hybrid PEDOT:Hep hydrogel and leads to the fast degradation. In contrast, hybrid PEDOT:PSS hydrogel is more stable, most likely caused by the synthetic PEDOT:PSS component. The overall faster degradation in vivo compared to the in vitro situation could also be caused by the mechanical stimulation induced by movements of the mice.

To evaluate the foreign body reaction of the mice to the injected materials, volumes of inguinal lymph nodes at hydrogel injection site and reference site without injection were determined by MRI (Figure 8A,C, and Figure S19C, Supporting Information). The volumes were compared to negative control (untreated) and positive control (inflammation induced by 12‐O‐tetradecanoylphorbol‐13‐acetate, TPA). Lymph nodes at the hydrogel injection sites tended to be larger than the negative control, but smaller than the positive control. Lymph nodes at the reference site were not enlarged. Among the three hydrogels, hybrid PEDOT:Hep hydrogel and GC/HA‐ALD hydrogel induced only minor increase of lymph node size, while hybrid PEDOT:PSS hydrogel caused relatively more lymph node enlargement.

Samples for histological analysis were prepared 11 d after the injections. At this time point, GC/HA‐ALD hydrogel has been completely degraded. Hybrid PEDOT:PSS hydrogel was degraded to 10% of the initial volume and hybrid PEDOT:Hep hydrogel was degraded, but left some dark spots in the tissue (Figure S19D,E, Supporting Information). The dark PEDOT component has not been fully degraded, though no intact hydrogel remained, as seen by MRI. No fibrous capsule formation was induced by the hydrogels, as capsule thickness around hybrid PEDOT:PSS hydrogel (154 ± 30 µm) was lower than negative control (245 ± 46 µm) (Figure S19E, Supporting Information). Study of specific tissue response showed an accumulation of macrophages (CD68+) around and in the hybrid PEDOT:PSS hydrogel, which mainly displayed alternative activation (CD206+) and, therefore, act in an anti‐inflammatory manner and support tissue repair (Figure 8E,F). GC/HA‐ALD hydrogel and hybrid PEDOT:Hep hydrogel did not induce macrophage accumulation at this time point, as their degradation has already been completed (Figure 8E,F). Accumulation of macrophages during hydrogel degradation has been observed previously.^37^ None of the three hydrogels induced an adverse inflammatory reaction (cyclooxygenase‐2 negative (COX‐2‐)), while no signs of matrix remodeling (transglutaminase‐2 negative (TG‐2‐)) and angiogenesis (vascular endothelial growth factor negative (VEGF‐)) were detected (Figure 8E,F).

Covalent crosslinking could improve the mechanical strength and cohesive force of a noncovalently assembled hydrogel; however, it could also impair various properties associated with a dynamic network, e.g., self‐healing, shear‐thinning, and injectability. In this work, we have developed a hybrid dynamic hydrogel system composed of noncovalent network and reversible imine crosslinking. The use of conductive polymers, not only led to electroconductivity, but also to a plethora of hydrophobic moieties, which are absent in most synthetic hydrogels. In many natural adhesives, such as proteins from burrowing ground frogs of the genus Notaden, sericin from the silkmoth *Bombyx mori*, and protein/saccharide associations from echinoderms, adhesion results from the synergy among different types of interactions, including hydrophobicity, electrostatics, and hydrogen bonds. The synthetic hybrid hydrogels present a rich source of positive and negative charges, hydrophobic/aromatic groups, and hydrogen bond donors/acceptors, while the dynamic network allows them to adapt and adhere to various surfaces. Moreover, the hybrid network of noncovalent and covalent crosslinking results in enhanced cohesion strength of the network, leading to adhesive materials with high mechanical stability.

In summary, we have created an electroconductive hybrid polymer network, in which both covalent and noncovalent crosslinking are reversible and dynamic. While the electroconductivity can function as an electronic sensor, as well as a biophysical cue to promote myogenesis, the cytocompatible adhesive can also be used to encapsulate cells for cell‐based therapy as injectable therapeutics. The materials were also characterized in vivo using immunocompetent mice, both for topical application and as injectable materials. The materials showed high biocompatibility in both studies. Interestingly, by replacing PEDOT:PSS with a polysaccharide‐doped bio‐electroconductive polymer PEDOT:Hep, the resulting hydrogel has shown remarkably enhanced adhesive strength on tissues, as well as fast degradation in vivo. These soft and stress relaxing materials can match the mechanical properties of soft tissues and would be particularly attractive for applications in heart, muscle, and neuron diseases, as well as wound dressings.

## 3 Experimental Section

Animal experiments were performed in accordance with the guidelines of German Regulations for Animal Welfare. The protocol was approved by the local Ethical Committee for Animal Experiments (reference number DD24.1‐5131/450/16). Details of the materials and experimental methods used are available in the Supporting Information.

## Conflict of Interest

The authors declare no conflict of interest.

## Supporting information

Supplementary

Supplementary

Supplementary

Supplementary

Supplementary

Supplementary

Supplementary

Supplementary

Supplementary

Supplementary

Supplementary

Supplementary
