# Supplementary material for: Cytocompatible, Injectable, and Electroconductive Soft Adhesives with Hybrid Covalent/Noncovalent Dynamic Network
Source: Adv Sci (Weinh). 2019 May 24;6(15):1802077. doi: 10.1002/advs.201802077 (PMC6685503; doi:10.1002/advs.201802077)
Supplement: Supplementary file 1 — Supplementary [file ADVS-6-1802077-s002.pdf]

Copyright WILEY-VCH Verlag GmbH & Co. KGaA, 69469 Weinheim, Germany, 2019.

ADVANCED SCIENCE
Open Access

Supporting Information

for *Adv. Sci.,* DOI: 10.1002/advs.201802077

Cytocompatible, Injectable, and Electroconductive Soft Adhesives with Hybrid Covalent/Noncovalent Dynamic Network

Yong Xu, Panagiotis A. Patsis, Sandra Hauser, Dagmar Voigt, Rebecca Rothe, Markus Günther, Meiying Cui, Xuegeng Yang, Robert Wieduwild, Kerstin Eckert, Christoph Neinhuis, Teuku Fawzul Akbar, Ivan R. Minev, Jens Pietzsch, and Yixin Zhang**

Supplementary Material for

Cytocompatible, injectable and electroconductive soft adhesives with hybrid covalent/non-covalent dynamic network

*Yong Xu, Panagiotis A. Patsis#, Sandra Hauser#, Dagmar Voigt, Rebecca Rothe Markus Günther, Meiying Cui, Xuegeng Yang, Robert Wieduwild, Kerstin Eckert, Christoph Neinhuis, Teuku Fawzul Akbar, Ivan R Minev, Jens Pietzsch*and Yixin Zhang**

---

* E-mail to: yixin.zhang1@tu-dresden.de and j.pietzsch@hzdr.de

This PDF file includes:

Material and Methods
Supplementary online text
Supplementary Figures S1 to S19
Caption for movies S1 to S9

Material and Methods

1 Materials

3,4-Ethylenedioxythiophene (EDOT) (Sigma-Aldrich, 483028), Ammonium persulfate (APS)(Sigma-Aldrich, A3678), Glycol chitosan(Sigma-Aldrich, G7753), Hyaluronic acid sodium salt(Sigma-Aldrich, 53747), Iron(II) sulfate heptahydrate(Sigma-Aldrich, 215422), Poly(sodium 4-styrenesulfonate)(Sigma-Aldrich, 243051), Sodium (meta) periodate (Sigma-Aldrich, S1878), Inc. Heparin, Sodium Salt, Porcine Intestinal Mucosa were obtained from EMD Millipore Corp., USA. PBS solution was prepared by dissolving a PBS powder in deionized water at pH 7.4. Gibco Dulbecco's Modified Eagle's Medium (DMEM, ThermoFisher, 10566-016), Fetal bovine serum (FBS, Sigma-Aldrich, F2442), Horse serum (Sigma-Aldrich, H1270), Penicillin-streptomycin (Sigma-Aldrich, P4333), LIVE/DEAD™ Viability/Cytotoxicity Kit were obtained from Life Technologies (Invitrogen, L3224). C2C12 cells (C3H muscle myoblast) were purchased from ECACC and cultured in DMEM supplemented with 10% (v/v) FBS in Petri dishes in a humidified incubator at 37 °C with 5 % under CO_2 atmosphere. Cardiac Troponin T antibody (Genetex, GTX28295), Phalloidin–Atto 633 (Sigma-Aldrich, 68825), Hoechst 33342 NucBlue® (Life Technologies, R37605, USA).

2 Methods

2.1 Synthesis and characterization of PEDOT:Hep and PEDOT:PSS

Heparin and PSS were used as counterions in the polymerization of EDOT. In a standard reaction, a 10-ml solution of Heparin or PSS (10% w/v in milliQ) was prepared at 25 °C, stirring under an argon gas ambiance, until completely dissolved (typically for 10 min). Then, EDOT was added dropwise to the solution and stirred for 10 min under argon ambiance, so that the mass ratio was 1.5:1 PEDOT:Heparin (or PEDOT:PSS). After that, a 2-ml solution of ammonium persulfate (APS) in milliQ (3:1 mass ratio to the heparin or PSS) was added dropwise, and stirred for 10 min under argon ambiance. Then, a solution of iron sulfate heptahydrate in milliQ (mass ratio 0.008:1 to the heparin) was added dropwise, and the solution was allowed to stir for 5 minutes under argon ambiance, and after that, milliQ was added until the final volume of 20 ml. The reaction was allowed to proceed for 72h or 48h at 4 °C. The samples were then centrifuged (5000 rpm, 10 min, 4 °C) and the supernatants were dialyzed against deionized water for 72h (molecular weight cutoff 10 kDa) and finally lyophilized and stored at 4 °C until further processing. Fourier-transform infrared (FTIR

NEXUS™ 870, Nicolet Instrument Corp., Madison, WI, U.S.) measurements were performed to obtain the infrared spectra of the samples, in order to validate the formation of bonds related to the reactions. UV-vis spectra were measured on a LAMBDA-35 UV-vis spectrophotometer (PerkinElmer, Inc., Waltham, MA, U.S.). The particle size of PEDOT:Heparin and PEDOT:PSS was characterized by dynamic light scattering (DLS) measurements with the use of analyzer Zetasizer Nano ZS (Malvern Panalytical Ltd., Malvern, UK). After the dialysis step of in the synthesis of PEDOT:Heparin and PEDOT:PSS, as described above, the samples were diluted 1:5 and 1:10 respectively in milliQ, to a concentration appropriate for the DLS measurements.

2.2 Synthesis and characterization of HA-ALD

For synthesis of HA-ALD, HA was first dissolved in 20 ml milliQ (5% w/v, typically for 1 h). A 600-μl solution of 0.5M sodium (meta) periodate in milliQ was added dropwise to the solution of HA to a final concentration of 0.015 M. The reaction was left to proceed 1h at RT, in the dark (as the sodium metaperiodate is light sensitive). Afterward the reaction is stopped by the addition of glycerol (0.05% v/v) for 2 h. Then the solution of oxidized hyaluronic acid was dialyzed against deionized water for 3 days (molecular weight cutoff 10 kDa) and finally lyophilized and stored at 4 °C until further processing. FTIR measurements were performed to obtain the infrared spectra of the samples, in order to validate the formation of the aldehyde functional groups and confirm the successful oxidization of HA.

2.3 Formation of electroconductive hydrogels

For the synthesis of the dual cross-linked hydrogels, solutions of HA-ALD, PEDOT:Heparin (or PEDOT:PSS) and GC in milliQ were mixed 1:1:2 at RT manually in microtubes. First the PEDOT:polymer solutions were mixed with the oxi-HA solutions 1:1 and the mixture was mixed with GC 1:1. Hydrogels with different concentrations of oxi-HA and PEDOT:polymer were synthesized.

2.4 Scanning electron microscopy (SEM)

Well-formed hydrogel samples were shock-frozen in liquid nitrogen and then freeze-dried by a lyophilizer to keep the original internal cross-linked structure of the hydrogel. Subsequently, the samples were covered with a gold powder coating of about 10 nm thickness (Emitech K550, Quorum Technologies Ltd, Ashford, UK), transferred to the SEM SUPRA 40VP-31-79 (Carl Zeiss SMT Ltd., Oberkochen, Germany) and examined at 5 kV accelerating voltage. SEM micrographs were taken using the software Smart SEM 05.03.05 (Carl Zeiss SMT Ltd., Oberkochen, Germany).

2.5 Hydrogel mechanical characterization

For the characterization of the mechanical properties of the hydrogels, rheological measurements were performed using a rheometer Anton-Paar MCR 302 (Anton Paar GmbH, Graz, Austria). The hydrogel samples were prepared directly on the surface of the rheometer plate by first adding the mixture of HA-ALD and PEDOT:Heparin (or PEDOT:PSS) and then, adding a GC solution (all in milliQ). The plate of the rheometer was then moved down, and brought in contact with the hydrogel. Different rheological tests were performed. The gelation process was followed by measuring the storage (G') and loss (G") moduli over time. The stress relaxation of the hydrogels was measured over time, after an initial shear strain of 1%. Amplitude and frequency sweeps were performed, where the moduli of the hydrogels were measured over a range of different strains (0.01% to 200%) and frequencies (0.01 Hz to 100 Hz), respectively. In continuous flow measurements the shear stress and viscosity of the hydrogels were monitored as a function of shear rate, in order to assess the shear-thinning behavior of the hydrogels. Finally, the self-healing behavior of the hydrogels was assessed by measuring the G' and G" at alternate step strain cycles of 1% and 800%.
The stiffness of the hydrogels was estimated by calculating the Young's modulus at a shear strain of 10%, using the

Equation 1:

$$E = 2 * G(1 + \nu) \;, \qquad G = \sqrt{G'^2 + G''^2} \tag{1}$$

, where *E* is the Young's modulus, *G* is the shear modulus, *G*' is the storage modulus, *G*" is the shear modulus and *v* is the Poisson's ratio. The Poisson ratio of the hydrogels was assumed to be 0.5 for this project, similarly to other studies on hydrogels**.**

2.6 Electrochemical measurements

For the cyclic voltammetry tests, a three-electrode setup was used with a working electrode, a reference electrode and a counter electrode (Fig. S6). A 30-μl volume of the hydrogels were formed around the working electrode, covering an electrode surface area of 0.008 cm^2 and were placed inside an electrolyte solution (PBS x1, pH 7.4). As the potential was applied to the working electrode (as a function of the stable potential of the reference electrode) a reduction or oxidation occurs and current begins to flow, which is recorded at the counter electrode. The potential that is applied at the working electrode was ramped linearly between the values -0.2 V and 1 V and the current was measured. The current density of the hydrogels was calculated by the ratio of measured current to the surface area of the electrode. For the Impedance testing, 50-μl volumes of hydrogels were placed between two glass carbon electrodes that were connected to an electrochemistry station (Biologic, SP-200, France). The

gap between the two electrodes was 5 mm and the diameter of hydrogel was 0.5 mm. The impedance of the hydrogels was recorded at 5 mV over a range of frequencies from 10^{-2} to 10^{5} Hz.

2.7 Conductivity testing

We used the 2 probe testing method to test the conductive of wet bulk gel, in order to exclude the effects of the ionic conductivity, the gels were formed with MilliQ dissolved GC, HA-ALD, and conductive polymers.

As shown in Figure S10B, we first do the Impedance testing, 50 µl volumes of hydrogels were placed between two glass carbon electrodes that were connected to the electrochemistry station (Biologic, SP-200). The gap between the two electrodes was 5 mm, and the diameter of hydrogel was 0.5 mm. The impedance of the hydrogels was recorded at 5 mV over a range of frequencies from 10^{-2} to 10^{5} Hz. The conductivity of the hydrogels was calculated using **Equation 2**[1,2]**:**

$$\delta = \frac{Re\,(Z)}{[Re\,(Z)]^2 + [Im\,(Z)]^{\,2}} * \frac{d}{S} \qquad (2)$$

, where *Re* (*Z*) and *Im (Z)* are the real and imaginary parts of the impedance (*Z*) at a frequency 1Hz, which is comparable to frequencies in physiological tissues [3]. *d* is the diameter and *S* is the area of the electrode.

2.8 Pull-off tests

The adhesion properties of the hydrogels was tested by pull-off measurements at a temperature of 24 ± 0.9 °C and a relative humidity of 54 ± 8.2%. Prior experiments, cover slides (9 mm diameter, silica glass, VWR International GmbH, Darmstadt, Germany) were cleaned by successive rinsing with soap water, acetone, ethanol, and milliQ, and drying with compressed air. One of these cover slides was fixed to a holder by double-sided adhesive tape. The other cover slide adhered to a FORT-25 force transducer (25 g capacity, World Precision Instruments Inc., Sarasota, USA) combined with a Lab-Trax-4/16 (LT4/16-5)-Transbridge 4M system (World Precision Instruments Inc., Sarasota, USA). The force transducer was attached to a motorized DC3314R micromanipulator with MS314 controller (World Precision Instruments, Sarasota, FL, USA), which was moved with the force transducer and the cover slide at constant speed of 20 µm * s^{-1}. The hydrogels were placed on the cover slide fixed to the holder. Then, the cover slide attached to the force transducer was brought in contact with 30 µl volume of the hydrogel sample situated on the cover slide fixed on the holder (without applying distinct preload). The thickness of the hydrogel layer was about 20 µm. Subsequently, the cover slides, and thus, the hydrogel in between them were pulled apart until

the hydrogel bond/sample separated/broke, mostly by cohesive failure. Using software LabScribe2 2.248000 (iWorx Systems Inc., Dover, U.K.), force-time curves were recorded, which were further transformed to force-distance curves to estimate the maximal traction force produced by a single pull-off runs of each test hydrogel.

The extension of hydrogel between cover slides during the pull-off tests was recorded with a custom-made 5-MP digital camera controlled by the AMCap video software. The adhesive strength (kPa) of the hydrogels was calculated as the ratio of the maximum pull-off force to the contact area of the hydrogels on cover slide at the initial force measurements.

2.9 Peeling tests

In addition, a double T-peeling test was performed using a universal test machine (Shimadzu, EZ-SX, Japan). Therefore, porcine cardiac tissue samples 15 mm (w) in width, 80 mm in length, 2 mm thick were adhered by the hydrogel from the same end, and the overlapping area was 15 mm × 40 mm. The other ends of both tissue samples were clamped, respectively (Figure S13C). Then, the upper tissue was pulled upward at a constant velocity of 10 mm*min^{-1} while the peeling force $\mathbf{F_p}$ was recorded. The interfacial toughness was calculated as $2\times\mathbf{F_p}/w$.

2.10 Lap-shear tests

Lap shear measurements were performed using a universal test machine (Shimadzu, EZ-SX, Japan) and the stress values were calculated by dividing the maximum shear force by the corresponding overlapping area of each sample. Four samples of each hydrogel mixing ratio (n=4) were used in the shear test. Fresh vacuum-packed porcine cardiac muscle tissue was commercially obtained from the supermarket and stored at 4 °C for later use. A small piece of fresh cardiac muscle was cut into rectangle sections at 20 mm × 20 mm using a razor blade. The excessive fat was shed off from the cut cardiac muscle pieces, and the thickness of tissues was controlled to be 2 mm, Then, one side of cardiac muscle was bonded to a glass slide (76 mm × 26 mm) with cyanoacrylate-based superglue and placed with the glass slide in PBS for immediate test in order to ensure the tissues were moist. An HA-ALD/conductive polymer mixture solution was applied on a piece of tissue and GC was introduced to trigger the formation of a thin layer of gel coating, onto which the other piece tissue was pressed immediately. The overlapping area was controlled to be 20 mm × 20 mm, and the samples were allowed to cure for 10 min at room temperature under humid condition. A tensile tester equipped with a 20 N load cell was used. The samples were fixed between the two film clamps and pulled apart at a velocity of 10 mm*min^{-1}.

2.11 Bioprinting

The Bioprinting tests were performed by an in-house custom built 3D printer (Figure 8, resolution: 200 µm). This printer had a custom linear guide, allowing the use of syringes as print heads. The hydrogels (0.2% PEDOT:Heparin, 0.5% oxi-HA, 1% GC) were preformed inside the syringe and extruded during the printing process. As a proof of principle a model of the B CUBE logo was printed (dark-colored in Figure 1J-K). The 3D printer was controlled by the Repetier-Host software and the 3D models (STL-files) were converted to code (G-Code) by the software Slic3r (http://slic3r.org/).

2.12 Conductive hydrogel degradation study

Hydrogel was prepared as described in the previous section. Four replicates were prepared in each group. The dry mass of freeze-dried electroconductive hydrogel were recorded (Mo). The gels were immersed in 1 mL PBS containing 4 mg * ml^{-1} hyaluronidase and 1 mg * ml^{-1} lysozyme and incubated at 37 °C. 50 µL of the buffer were collected and replaced by fresh enzyme solution until to 21 days after the initial setup. At predetermined time points, the samples were removed, and mass (Mt) of freeze-dried samples was recorded. The percentage of mass loss was calculated according to the following equation: % mass = [(Mo-Mt)/ M_o] * 100%.

2.13 Tissue adhesive testing

The chicken heart were obtained from slaughterhouse waste (not older than 3 days after slaughter), and cleaned with 70 % ethanol and Milli-Q water for three times before doing the testing. The hydrogels (0.5% PEDOT:Hep, 0.5% HA-ALD, 1% GC, Size: circular shape, diameter: 2.5 cm, thickness: 0.5 mm) were nicely attached on the chicken heart and incubated in a plastic wetting bag for overnight, then to test if the hydrogel remains adhered to the muscle tissue under flushing with water and or under the water with stirring (240 rpm / min) (Movie S7,S8).

2.14 Living animal adhesion testing

Adhesion of hydrogel 0.5% HA-ALD + 1% GC + 0.2% PEDOT:PSS on mouse skin was tested by squeezing hydrogel out of the syringe directly on the lower back of the mouse, and removing hydrogel after 30 min with the help of a tweezer. Adhesion of hydrogel during this time period was documented by photos and videos (Figure S11, Movie S9).

2.15 C2C12 cell culture in the hydrogel

2.15.1 Culturing of C2C12 cells

A vial of frozen C2C12 cell line (ECACC) was thawn in a 37 °C water bath for 2 minutes. The cells were transferred to the 5-ml full cell culture medium (DMEM GlutaMax low

glucose with 10% FBS), After mixing, the suspension was transferred into a 15 ml falcon tube and centrifuged at 1000 rpm * min^{-1} for 3 minutes. The supernatant was discarded and the cell pellet suspended in 7-ml full cell culture medium. The cell suspension was transferred to a T-25 cell culture flask and incubated at 37 °C, 95% humidity and 5% CO_2. To preserve the myoblast characteristics, the passage of the cells was performed at cell confluency between 50% and 60%.

2.15.2 Culturing C2C12 cells in the hydrogel

10 µL GC (contains 2×10^3 cells) and 10 µl HA-ALD with PEDOT:heparin or PEDOT:PSS mixture were mixed in a µ-Slide (Ibidi, Uncoated, Germany), then incubated at in the incubator for 20 min, then washed two times with DMEM medium (10% FBS,10 µL), then covered with DMEM medium (10% FBS, 20 µL), and put it into the incubator (37 °C and 5% CO_2).

2.15.3 Live-Dead cell staining

The C2C12 cells were seeded into the hydrogel in µ-Slide (Ibidi, Uncoated, Germany), and DMEM medium (10% FBS, 10 µL) was added. After 1 day, 3 days and 7 days after the initial setup of culturing, the resulting approximately 2 µM calcein AM and 4 µM EthD-1 working solution was added directly to cells and incubated at 37 °C for 1 h. Then, the wells were observed with a confocal laser scanning microscope Zeiss LSM 780 (Carl Zeiss Microscopy GmbH, Jena, Germany).

2.15.4 Differentiation of C2C12 cells and immunofluorescence staining

For the differentiation studies, the C2C12 cells were cultured on the hydrogels at a cell density of 2000 cells per well in a µ-slide (Ibidi, Uncoated, Germany). When cells achieved 50% confluence, the 10% FBS DMEM medium was replaced with low-glucose DMEM supplemented with 2.5% horse serum (Sigma-Aldrich) and 1% penicillin/streptomycin in order to induce cell differentiation. Then, 3 days and 7 days after the initial setup of culture in differentiation medium, the cells were fixed with 4% PFA for 1h. Cells were then permeabilized with 0.1% Triton X-100 (Sigma-Aldrich, T9284) for 20 minutes at 25 °C, followed by blocking with 3% BSA for 12 hours under 4℃. Subsequently, cells were incubated with phalloidin for 30 minutes or exposed to immunofluorescent staining. Therefore, samples were incubated with primary mouse anti-Cardiac Troponin T antibodies diluted at a ratio of 1:400 (Genetex, GTX28295) for 2 hours. Secondary antibodies, anti-mouse Alexa Fluor 647 (Invitrogen, A-21235) were applied at a ratio of 1:500 for 1 hour and nuclei were counter-stained with Dapi Hoechst 33342 (1:4000). Then, samples were imaged with LSM.

To evaluate the differentiation abilities of these cells cultured on the hydrogels, quantitative analysis of the myogenic C2C12 differentiation was performed by calculating the fusion index, the troponin T-positive myotubes area, number, and the myotubes length. The fusion index corresponds to the percentage of the number of nuclei within the multinucleated myotubes to the total number of nuclei. Four images of each condition were analyzed using an image processing software (ImageJ, National Institutes of Health, Bethesda, MD, USA).

2.16 In vivo biocompatibility testing

2.16.1 Hydrogel injection

Animal experiments were performed in accordance with the guidelines of German Regulations for Animal Welfare. The protocol was approved by the local Ethical Committee for Animal Experiments (reference number DD24.1-5131/450/16). Hydrogels were injected as described previously.[4] Briefly, female immunocompetent SKH1-Elite mice were purchased from Charles River. Mice (age 17 weeks, weight 25–30 g) were anesthetized using 8% (v/v) desflurane (Baxter) and were subcutaneously injected with 50 µl hydrogel (either HA-ALD + GC, HA-ALD + GC + PEDOT:PSS or HA-ALD + GC + PEDOT:Hep) in the lower back area (Figure S18). Hydrogels were gelated for 5 min before injection.

2.16.2 In vivo volume determination measurements

Volumes of injected hydrogels and inguinal lymph nodes were measured using dedicated 7T small animal magnetic resonance imaging (MRI, Bruker) with a T2-weighted measuring sequence (TRARE) (Figure S19B). Echo and repetition time was 38 ms and 5774 ms, respectively. Spatial resolution was 150 µm in xy-direction and 800 µm in z-direction. Quantification of the hydrogels and lymph node volumes was performed using the software ROVER (ABX GmbH).

2.16.3 Histological analysis

Endpoint histological analysis was performed at day 11 after injection of the hydrogels as described previously.[5] Briefly, three animals per group were sacrificed and hydrogels were removed including the surrounding tissue. Tissue samples were fixed in 4% (v/v) PFA for 24 h and in 20% (w/v) sucrose in PBS for 3 d. For cryosection, samples were embedded in 7.5% (w/v) gelatin in 20% (w/v) sucrose solution in PBS, frozen, and cut to 5 µm sections in a cryostat at 30°C. Hematoxylin & eosin (H&E) stain and Van-Gieson’s stain to measure capsule thickness were performed as described previously using standard protocols.[6] Measurement of capsule thickness was performed using Axio Vision software (Carl Zeiss). For each section 5 points on each capsule site (skin and muscle site) of the remaining

hydrogel were measured (Figure S19 A). Specific tissue response was visualized using immunohistological stainings for CD68 (pan macrophages), CD206 (M2 macrophages), cyclooxygenase-2 (COX-2, inflammation), transglutaminase-2 (TG-2, matrix remodeling), and vascular endothelial growth factor (VEGF, angiogenesis) as described previously.[5] After antigen retrieval in 10 mM heated citrate buffer for 20 min (except for CD68), quenching of endogenous peroxidase in 3% (v/v) hydrogen peroxide for 10 min and quenching of endogenous biotin using Biotin-Blocking System from Dako according to manufacturer's instructions was performed. Unspecific binding was blocked by incubation in 10% (v/v) FCS in PBS for 1 h before tissue sections were incubated with primary antibody (anti-CD68, MCA-1957, AbD Serotec, 1:100, rat; anti-CD206, ab64693, Abcam, 1.100, rabbit; anti-COX-2, ab15191, Abcam, 1.500, rabbit; anti-TG-2, sc20621, Santa Cruz Biotechnology, 1:50, rabbit; anti-VEGF, sc152, Santa Cruz Biotechnology, 1:100, rabbit) or isotype control (normal rat IgG, sc-2026, Santa Cruz Biotechnology; rabbit polyclonal IgG, ab27478, Abcam; concentrations comparable to primary antibody) over night. Sections were incubated with biotinylated secondary antibody against rabbit (IgG-biotinylated, 111-065-003, Dianova, 1:200, goat) or rat (IgG-biotinylated, 312-066-045, Dianova, 1:200, rabbit) for 1 h, which was visualized by incubation with ExtrAvidin peroxidase (Sigma Aldrich) for 30 min and AEC substrate kit (BD Biosciences) for 2 – 5 min. Sections were counterstained with Mayer's hematoxylin and embedded in aqueous solution. Sections were imaged using AxioImager.A1 microscope and AxioVision software (Carl Zeiss). Quantification of immunohistological stainings were performed using ImageJ/FIJI. Color threshold plugin was used, and RGB values were set for cell nuclei and immunohistological positively stained areas. After applying the analyze particles plugin, positively stained area was divided by cell nuclei area.

2.16.3 Histological statistical analysis

Statistical significance of hydrogel and inguinal lymph node volume over the time course, as well as for histological investigations was calculated using a one-way ANOVA followed by a Bonferroni post hoc test using Prism 7 (GraphPad Software). Statistical significance was assumed for $p < 0.05$, $p < 0.001$ for all statistical analyses.

2.17 Statistical Analysis

Unless otherwise noted, all experiments were performed in triplicate ($n = 3$) and data are presented as mean ± s.d. Statistical analysis was performed using OriginPro 2015G (OriginLab Corp.) by using the one-way ANOVA followed by Tukey multiple pairwise comparison tests, P-values < 0.05 were considered as statistically significant.

Supplementary Figures

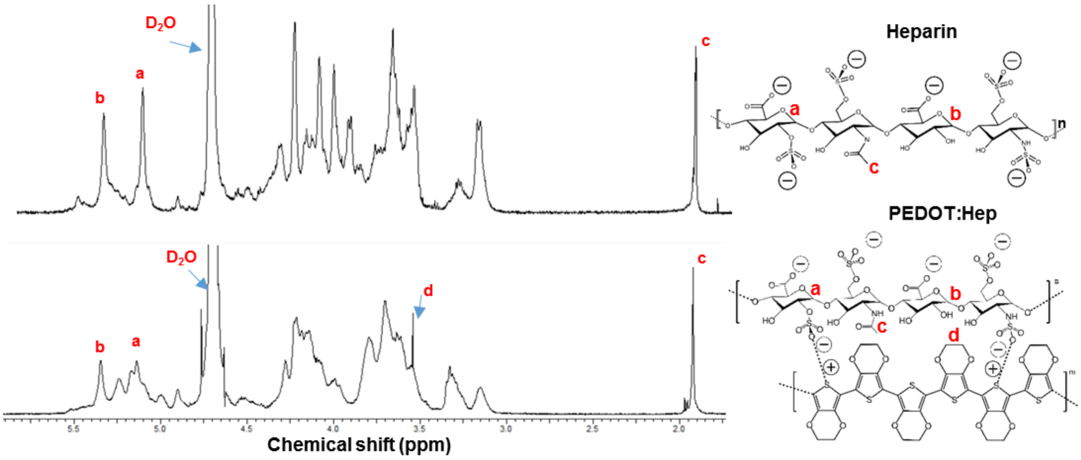

Figure S1: Proton nuclear magnetic resonance (^{1}H-NMR) spectra of heparin and PEDOT:Hep. The signals of a, b indicates anomeric protons of heparin and c indicates the acetyl group of N-acetyl-D-glucosamine in the molecular structure of heparin, d indicates the -O-CH_2- protons of EDOT units.

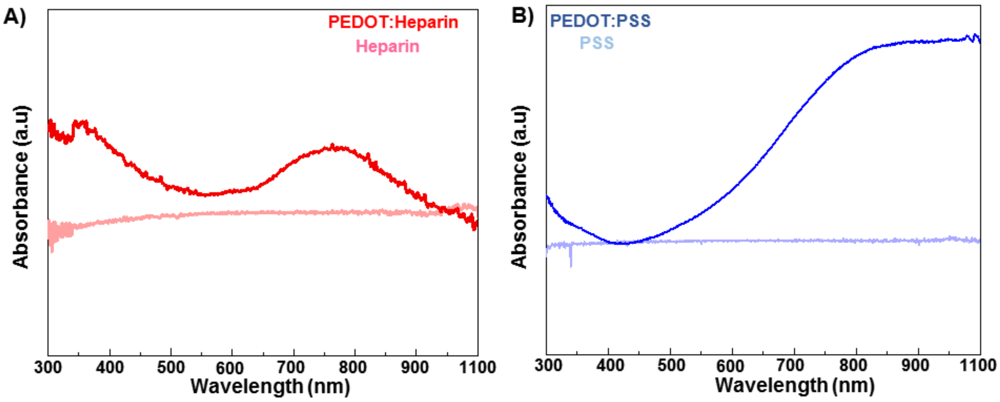

Figure S2: Spectrophotometric detection of the EDOT polymerization on PSS and Heparin. (A) Absorbance spectra of PEDOT:Hep and Heparin solutions in MilliQ water. (B) Absorbance spectra of PEDOT:PSS and PSS solutions in MilliQ water.

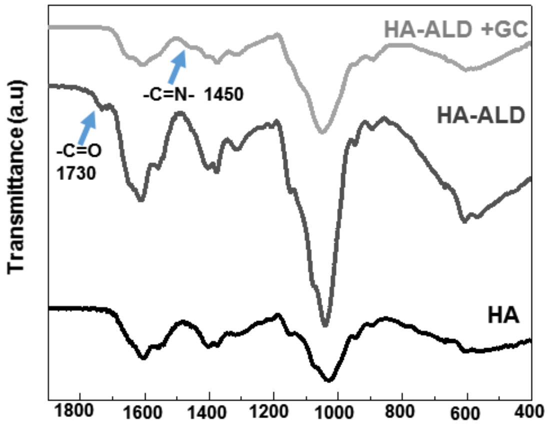

Figure S3: Fourier Transform Infrared (FT-IR) spectra of HA, HA-ALD and HA-ALD+GC. The peak at 1730 cm^{-1} corresponds to the aldehyde groups in the HA-ALD sample. The peak at 1450 cm^{-1} corresponds to a Schiff base (-C=N-), which is formed between the aldehyde groups of HA-ALD and the amine groups of GC.

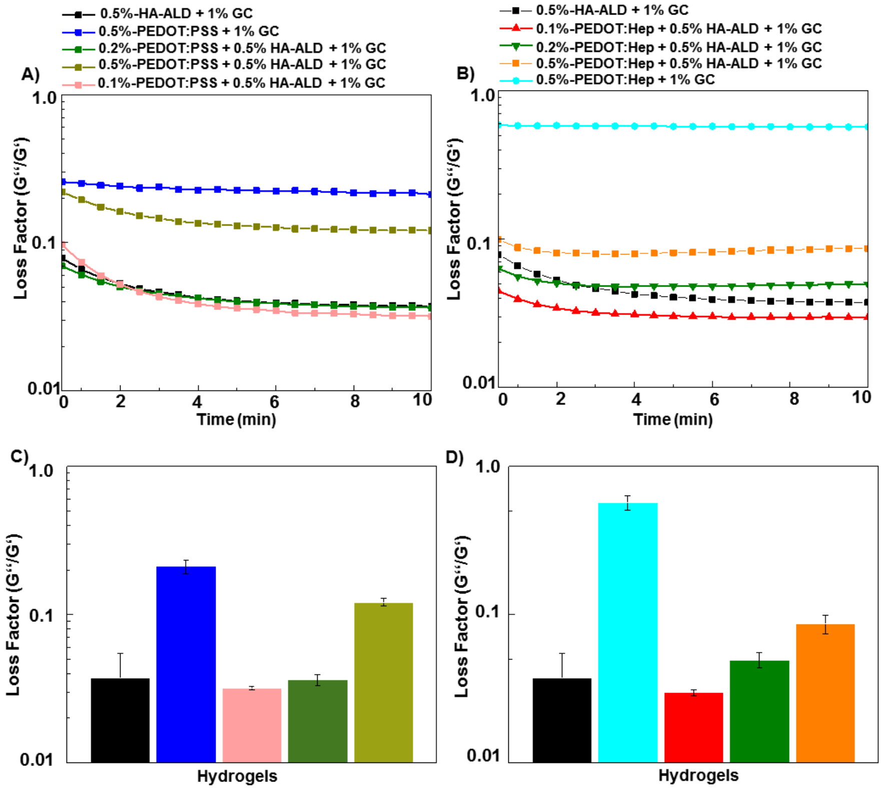

Figure S4: The loss factor of the hydrogel samples. (A) PEDOT:PSS hydrogels (the loss factor plotted against the time). (B) PEDOT:Hep hydrogels (the loss factor plotted against the time). (C) The loss factor at 10 min of gelation for hydrogels with PEDOT:PSS. (D) The loss factor at 10 min of gelation for hydrogels with PEDOT:Hep. Columns represent as means, error bar, n=3.

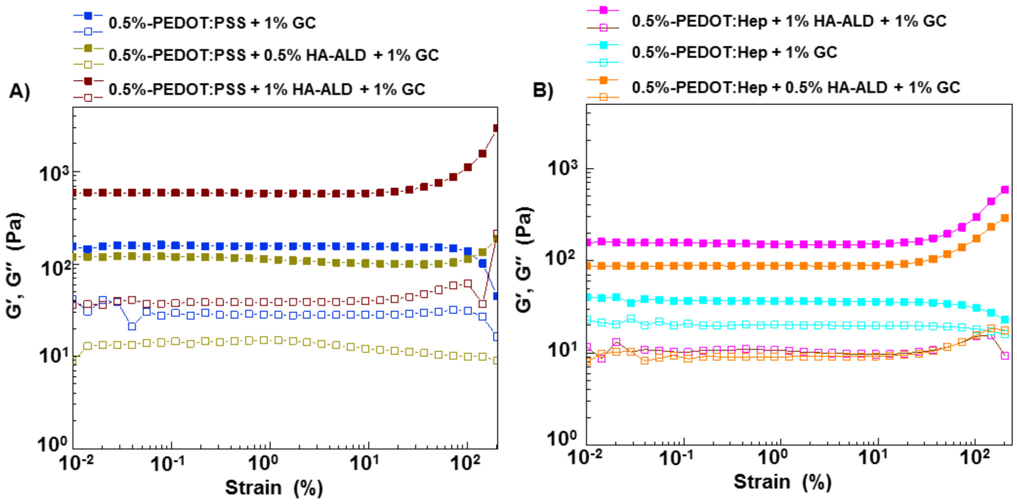

Figure S5: Amplitude sweep performed at a constant frequency of 1% and the shear strain increasing from 0.01% to 200%. (A) Amplitude sweep of PEDOT:Hep hydrogels. (B) Amplitude sweep of PEDOT:PSS hydrogels.

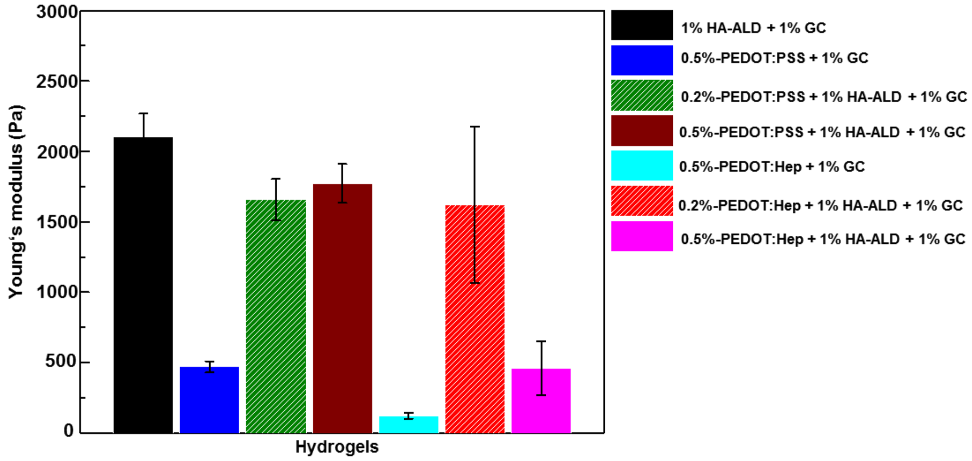

Figure S6: The Young's modulus of hydrogels with 1% HA-ALD, at a 10% shear strain. Columns represent as means, error bar, n=3

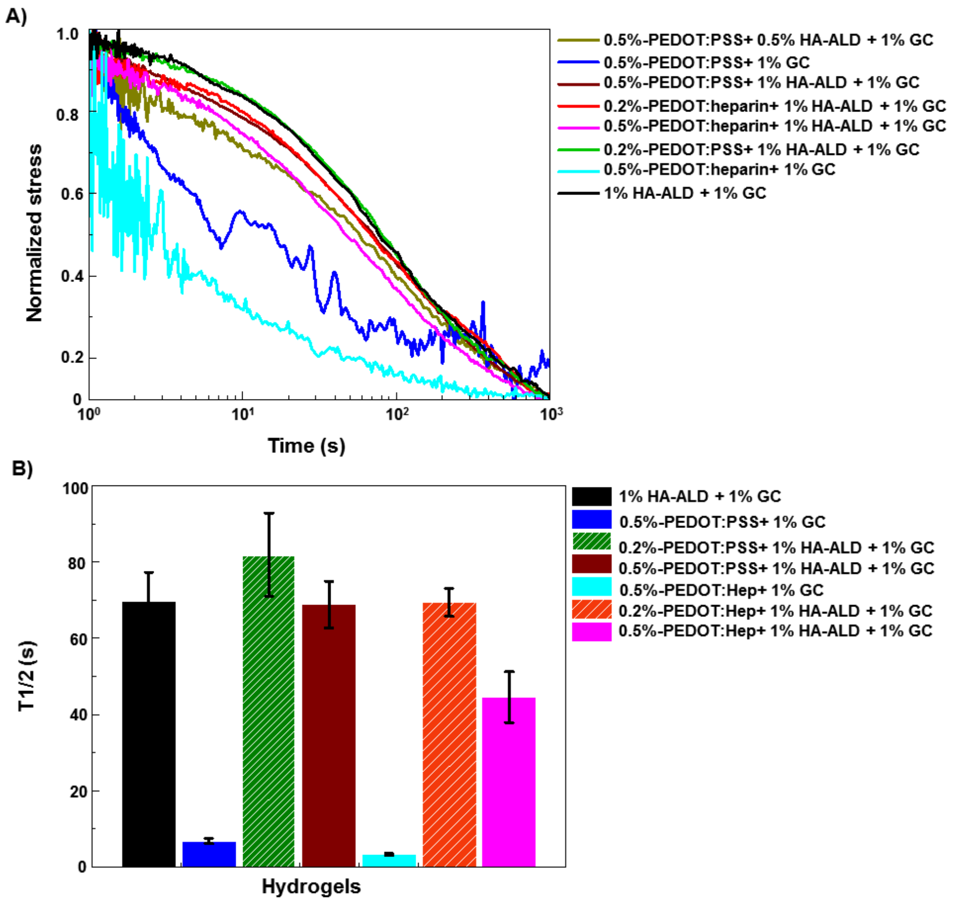

Figure S7: Stress relaxation test of the hydrogels at a constant shear strain (1%). (A) The stress plotted against time of hydrogels with PEDOT:Heparin. (B) The stress relaxation half-times (τ1/2) of PEDOT:Heparin hydrogels. Columns represent as means, error bar, n=3

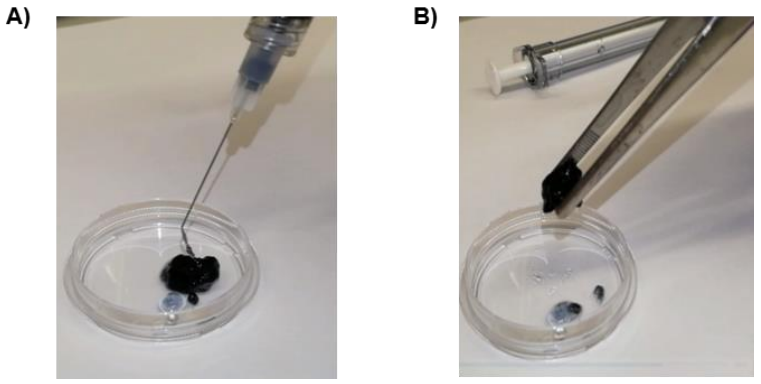

Figure S8: (A) Injection of a hydrogel via a syringe with 27G needle onto a plastic plate. (B) The injected hydrogel maintains its structural integrity when gripped and lifted with tweezers.

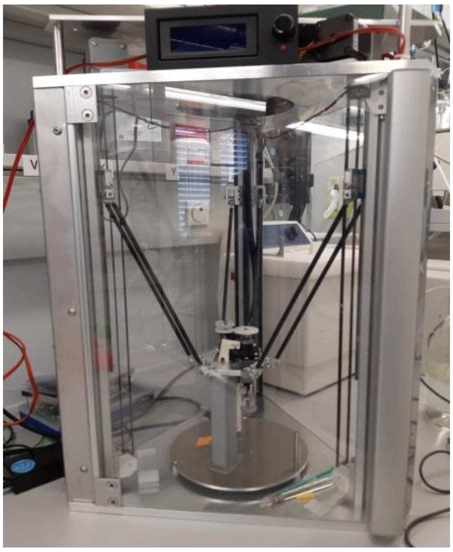

Figure S9: The image of the custom-made 3D printer.

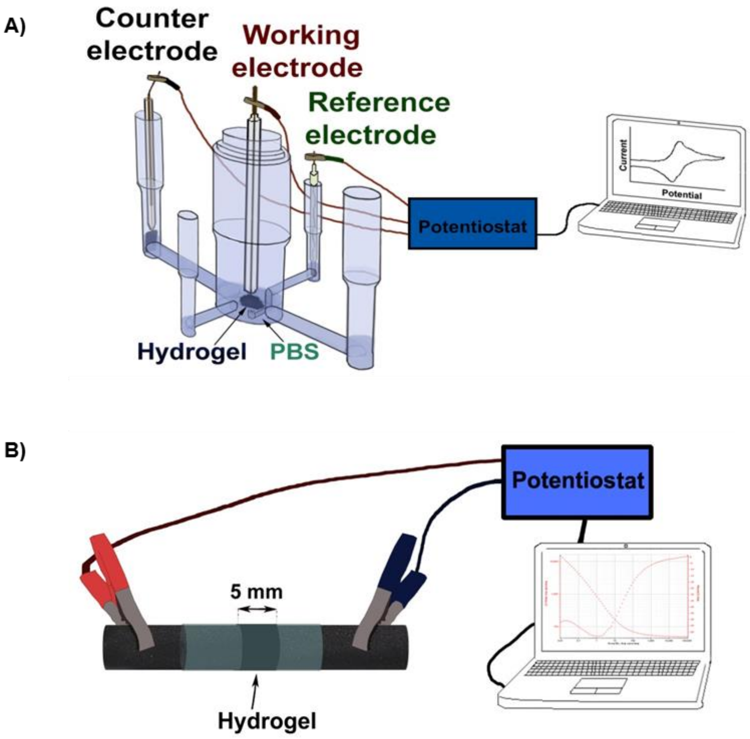

Figure S10: (A) Scheme of the experimental setup of the cyclic voltammetry tests. (B) Scheme of the experimental setup of the impedance measurements.

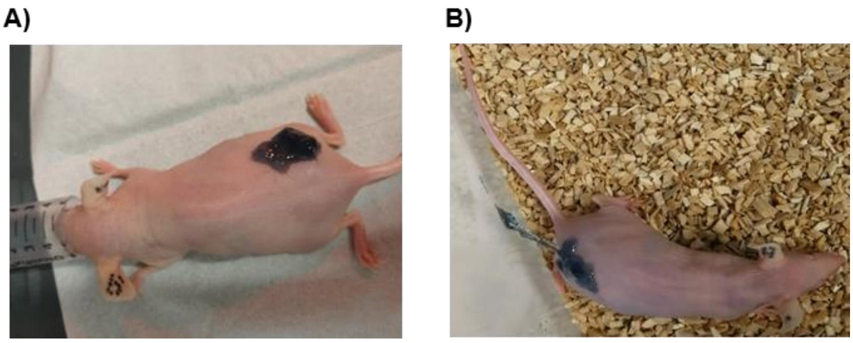

Figure S11: Hydrogel adherence on a living naked mouse. (A) The black-coloured conductive hydrogel (0.2% PEDOT:PSS + 0.5% HA-ALD + 1% GC) attached to the back of female immunocompetent SKH1-Elite mouse under narcosis. (B) The hydrogel maintains the stable adhesion on the living mouse when it was running in the feeding box. The gel was first attached on the mouse back and then partially attached the wall because of the random running of the mouse in the box. The gel is stretched because the adhesion of the gel on the mouse skin and the plastic surface.

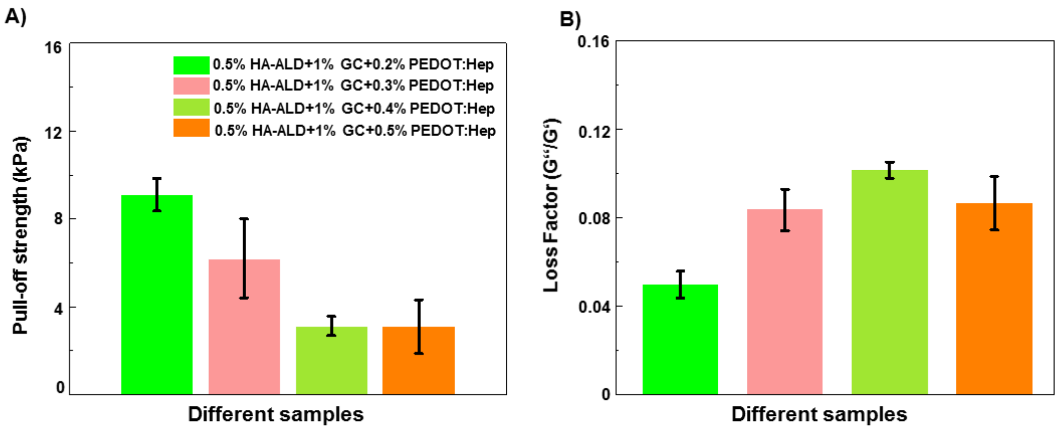

Figure S12: (A) Pull-off strengths of conductive hydrogels with different percentages of PEDOT:Hep. (B) The loss factors of conductive hydrogels with different percentages of PEDOT:Hep.

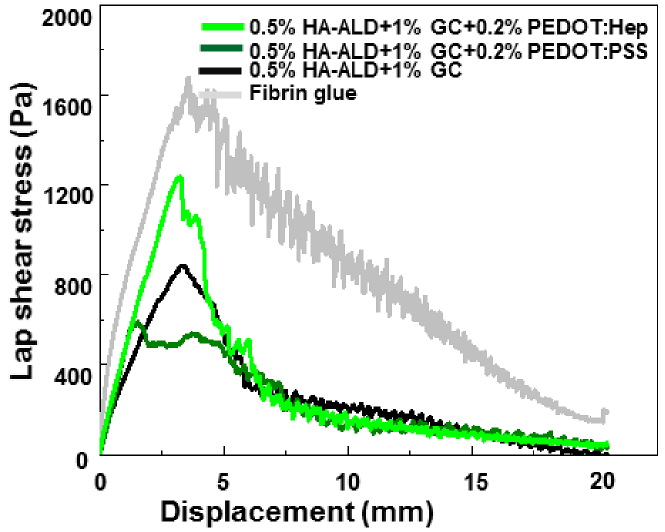

Figure S13: Lap shear test. Lap shear stress and displacement curve (n=4).

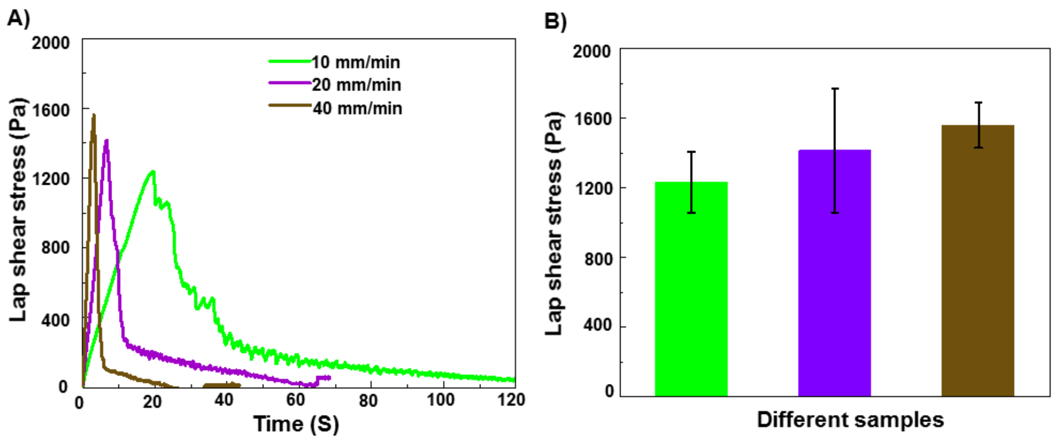

Figure S14: lap-shear test with different shear speeds. (A) Adhesion mechanical curves in lap-shear testing with conductive hydrogel-glued porcine myocardium tissue (0.2% PEDOT:Hep + 0.5% HA-ALD + 1% GC) at different shear speeds and (B) adhesive strength of conductive hydrogel measured at different shear speed (n =

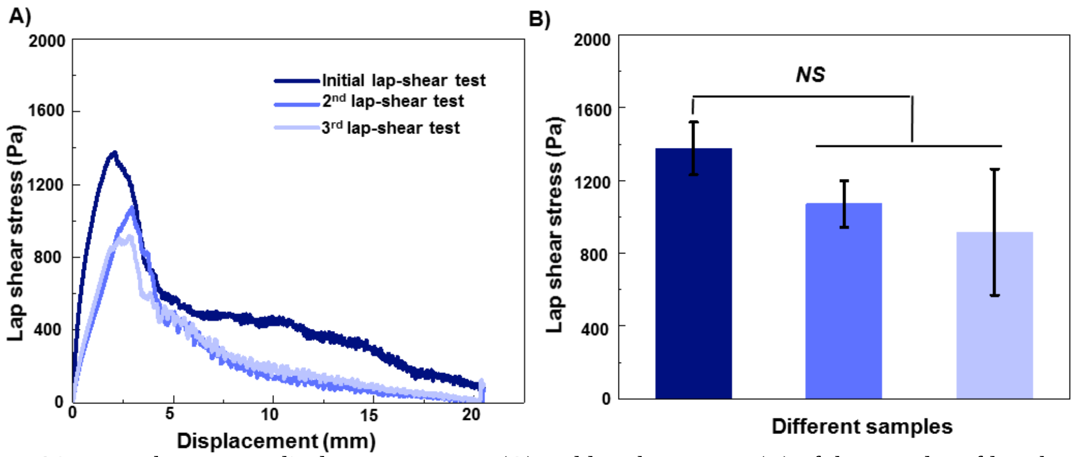

Figure S15: Lap shear stress-displacement curves (A) and lap shear stress (B) of three cycles of lap shear testing of conductive hydrogel (0.2% PEDOT:Hep + 0.5% HA-ALD + 1% GC) on porcine myocardium tissue. Statistically significant differences are shown with asterisks * $p<0.05$, ** $p<0.01$, *** $p<0.001$, mean, error bar, n=4.

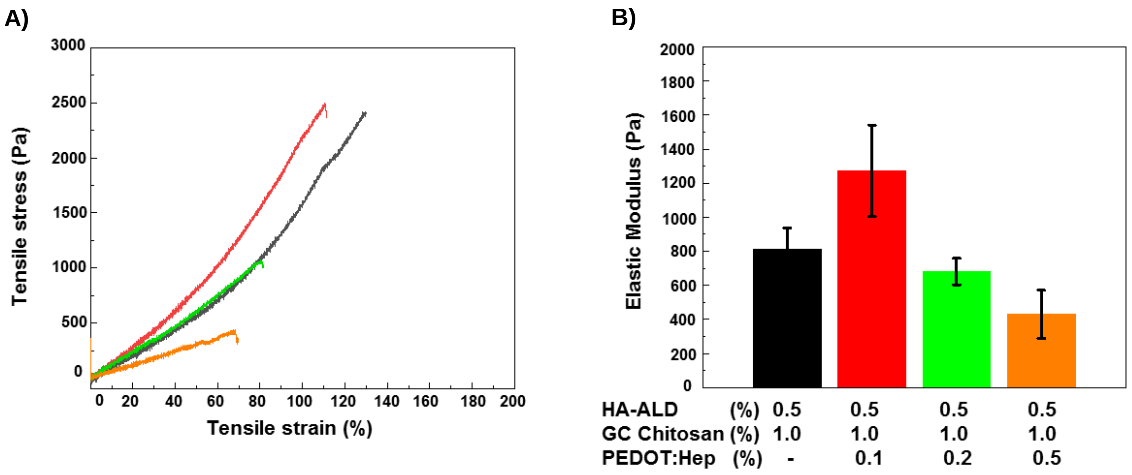

Figure S16: Tensile stress and elastic modulus of hydrogels. (A) Stress-strain curve. (B) Young's modulus (mean, error bar, n=3).

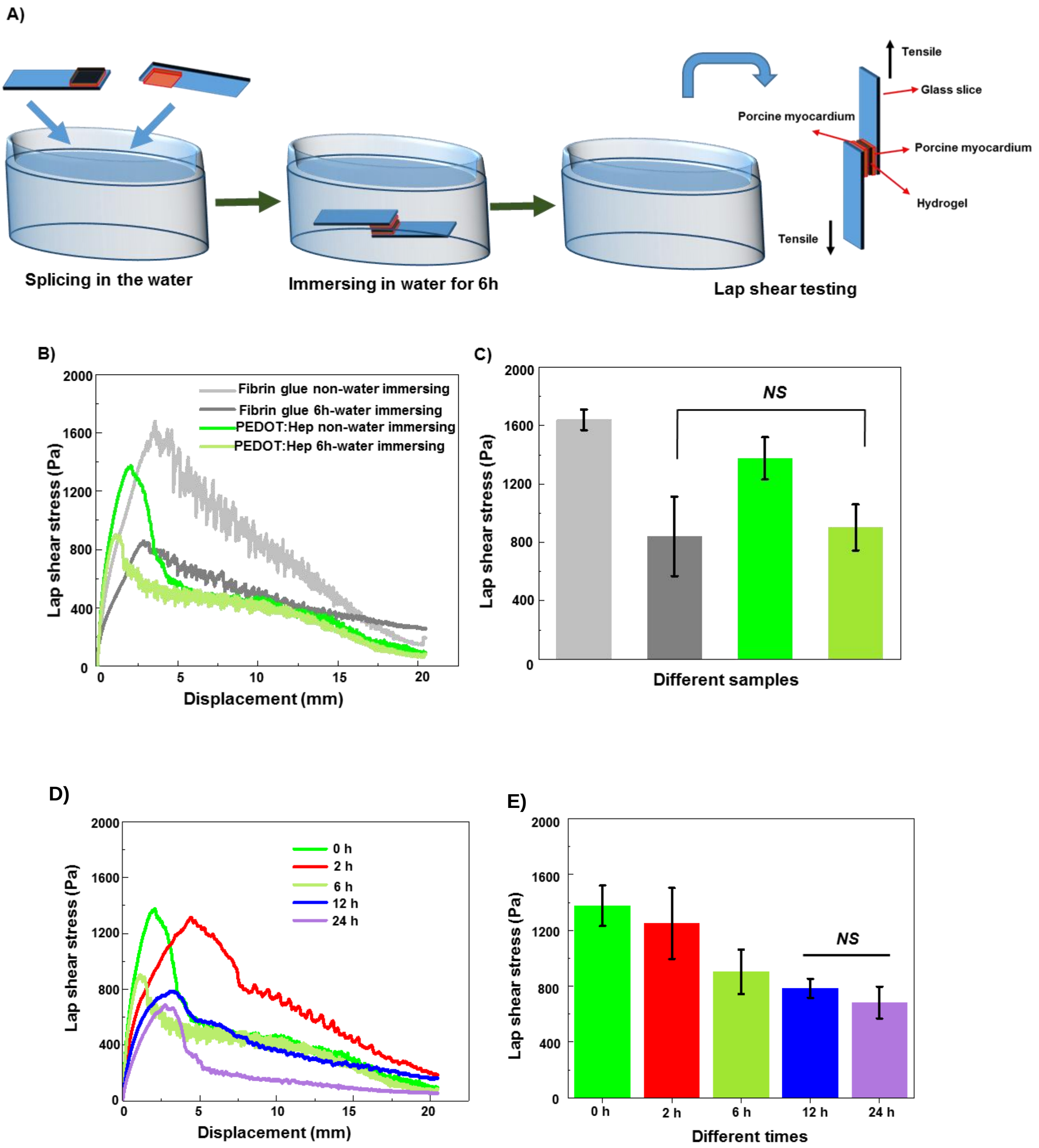

Figure S17: (A) Schematic drawing of under-water treatment of conductive hydrogel and fibrin glue. (B) Adhesion mechanical curves of conductive hydrogel and fibrin glue measured in shear tests (fibrin glue and 0.2% PEDOT:Hep + 0.5% HA-ALD + 1% GC) on porcine myocardium tissue before and after 6 hour under-water treatment. (C) Shear stress of hydrogels and fibrin glue before and after under-water treatment (mean, error bar, n=4). (D) Lap shear stress-displacement curves of conductive hydrogel measured in shear tests (0.2% PEDOT:Hep + 0.5% HA-ALD + 1% GC) on porcine myocardium tissue before and after under-water treatment with different times. (E) Shear stress of hydrogels before and after under-water treatment (mean, error bar, n=4).

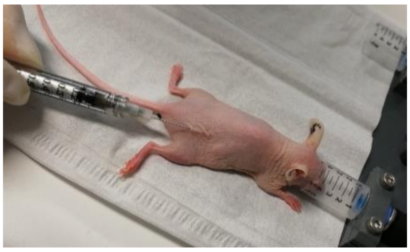

Figure S18: The picture to show the injection of conductive hydrogels.

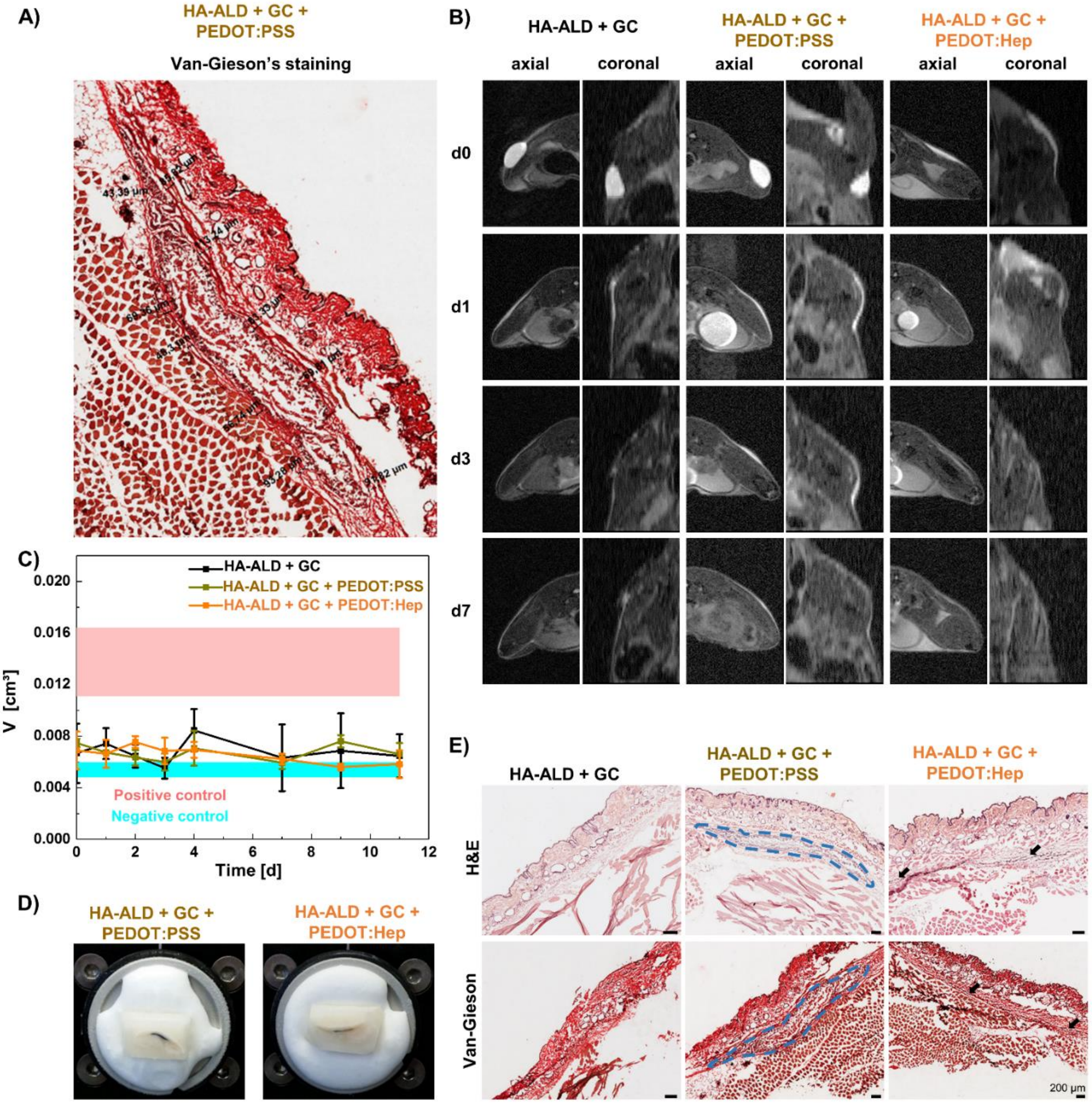

Figure S19: **In vivo evaluation of hydrogel degradation and biocompatibility.** (A) Representative determination of capsule thickness, (B) Representative MRI images used for volume determination of hydrogels (C) Volume determination of inguinal lymph nodes at sites without hydrogel injection by MRI, compared to negative (untreated) and positive (TPA injection) control, n = 3; Mean ± SD; one-way ANOVA, Bonferroni *post-hoc* test, (D) Photographs of tissue samples used for histological investigations, (E) Representative histological images of H&E and Van-Gieson's stain. Blue line indicates hydrogel-tissue interface and black arrows highlight remaining dark PEDOT.

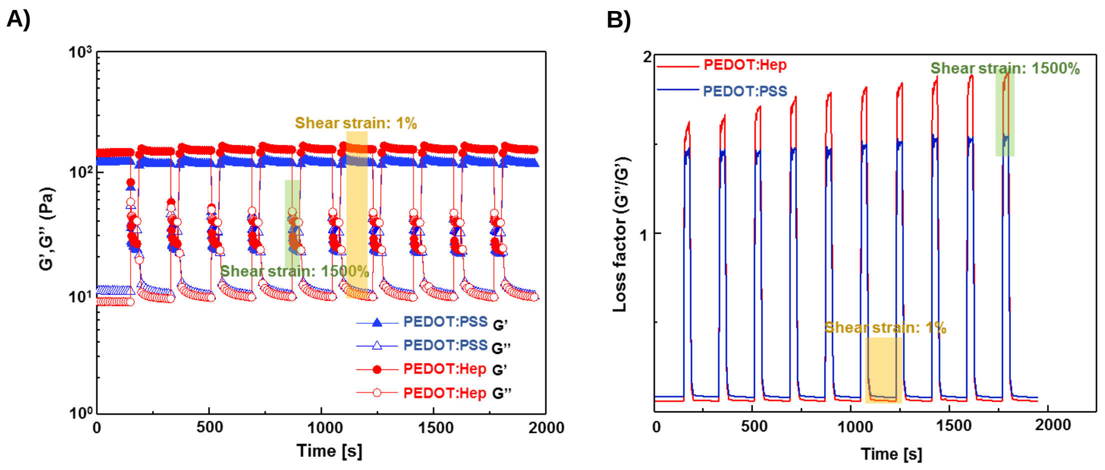

Figure 20: Self-healing capability detected by rheological measurements. (A) The alternate step strain was switched from 1% to 1500% within 10 cycles. (B) The change of loss factor related to alternate step strain.

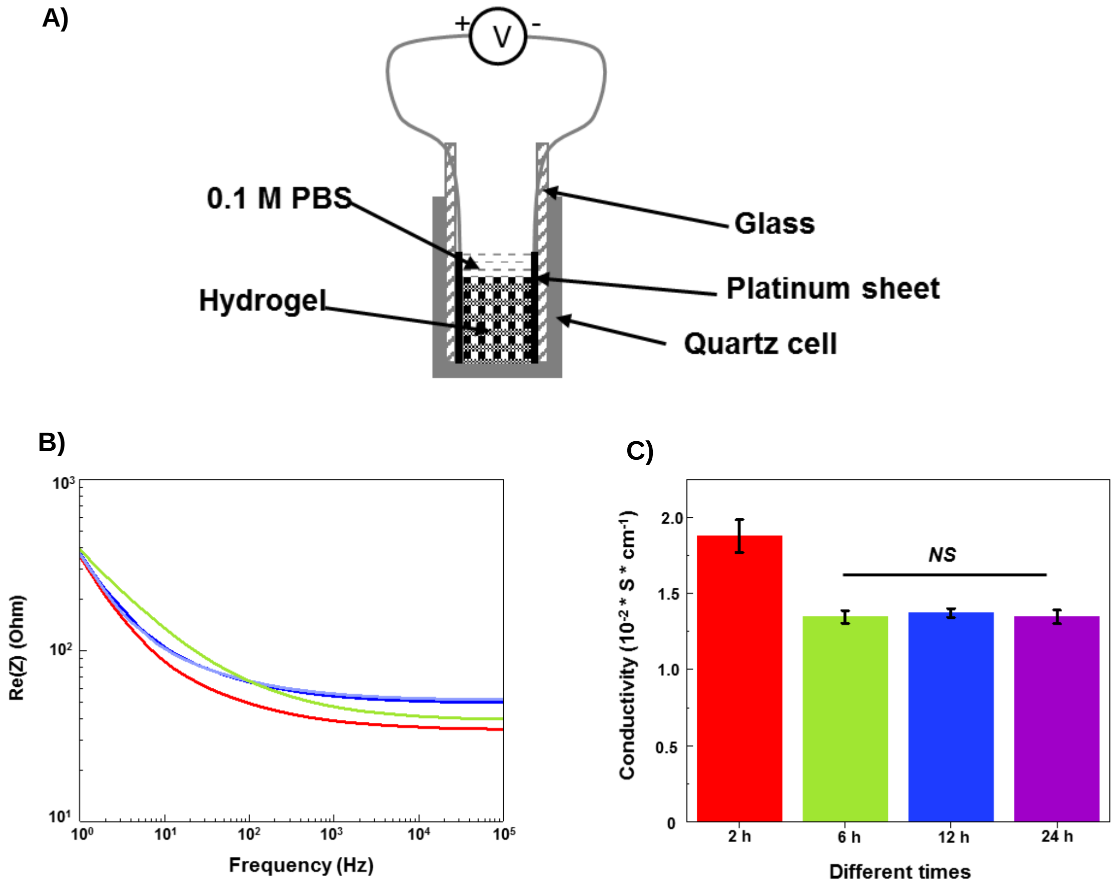

Figure 21: (A) Schematic diagram displaying the custom-made platinum sheet sandwich electrode. (B) Electrochemical impedance spectroscopy (Z vs frequency) of hydrogels with different time periods of immersing in PBS buffer. (C) The conductivity of hydrogels after different PBS immersing time (means, error bars, n=3).

Caption for Movies

Movie S1. Injection of electroconductive hydrogel (0.2% PEDOT:Hep + 1% GC + 0.5% HA-ALD) in the MilliQ warter.
Movie S2. Injection of electroconductive hydrogel (0.2% PEDOT:PSS + 1% GC + 0.5% HA-ALD) in the MilliQ warter.
Movie S3. Injection electroconductive hydrogel (0.2% PEDOT:PSS + 1% GC + 0.5% HA-ALD) via a syringe with 27G needle onto a plastic plate. Then the injected hydrogel maintains its structural integrity when gripped and lifted with tweezers.
Movie S4. Bioprinting of electroconductive hydrogel (0.2% PEDOT:Hep + 1% GC + 0.5% HA-ALD)**.**
Movie S5. Pull off testing of PEDOT:Hep + GC + HA-ALD formed electroconductive hydrogel.
Movie S6. Pull off testing of PEDOT:PSS + GC + HA-ALD formed electroconductive hydrogel.
Movie S7. Adhesion of the electroconductive hydrogel (0.2% PEDOT:Hep + 0.5% HA-ALD + 1% GC) on muscle tissue (chicken heart), and the hydrogel remains adhered to the muscle tissue under flushing with water.
Movie S8. Adhesion of the electroconductive hydrogel (0.5% PEDOT:Hep 0.5% HA-ALD 1% GC) on muscle tissue (chicken heart), the hydrogel remains adhered to the muscle tissue under water with the shaking of the water tank.
Movie S9. Adhesion of the electroconductive hydrogel (0.2% PEDOT:PSS 0.5% HA-ALD 1% GC) on the skin of living mice.
Movie S10. Bioprinting of electroconductive hydrogel (0.2% PEDOT:Hep + 1% GC + 0.5% HA-ALD) on the porcine skin.
Movie S11. The porcine skin is is immersed into the water , submersed it for three times after bioprinting, and the printed 4-layers scaffold kept intact and adhered tightly to the tissue.

Movie Linkage:

https://cloudstore.zih.tu-dresden.de/index.php/s/F14NKYpI5XsN3ai

References

[1] V. P. Orlovskii, V. S. Komlev, S. M. Barinov, *Inorg. Mater.* **2002**.

[2] R. P. Suvarna, K. R. Rao, K. Subbarangaiah, *Bull. Mater. Sci.* **2002**.

[3] A. F. G. Godier-Furnémont, M. Tiburcy, E. Wagner, M. Dewenter, S. Lämmle, A. El-Armouche, S. E. Lehnart, G. Vunjak-Novakovic, W. H. Zimmermann, *Biomaterials* **2015**.

[4] C. Tondera, R. Wieduwild, E. Röder, C. Werner, Y. Zhang, J. Pietzsch, *Adv. Funct. Mater.* **2017**, *27*.

[5] S. Ullm, A. Krüger, C. Tondera, T. P. Gebauer, A. T. Neffe, A. Lendlein, F. Jung, J. Pietzsch, *Biomaterials* **2014**.

[6] C. Tondera, S. Hauser, A. Krüger-Genge, F. Jung, A. T. Neffe, A. Lendlein, R. Klopfleisch, J. Steinbach, C. Neuber, J. Pietzsch, *Theranostics* **2016**.
